# PPIL2 is a target of the JAK2/STAT5 pathway and promotes myeloproliferation via degradation of p53

**DOI:** 10.1172/JCI181394

**Published:** 2025-05-08

**Authors:** Pan Wang, Xu Han, Kehan Ren, Ermin Li, Honghao Bi, Inci Aydemir, Madina Sukhanova, Yijie Liu, Jing Yang, Peng Ji

**Affiliations:** 1 Department of Pathology, Feinberg School of Medicine and; 2 Robert H. Lurie Comprehensive Cancer Center, Northwestern University, Chicago, Illinois, USA.

**Keywords:** Cell biology, Hematology, Bone marrow, Cancer

## Abstract

The activated JAK2/STAT pathway is characteristic of myeloproliferative neoplasms (MPNs). The pleckstrin 2 (PLEK2) signalosome is downstream of the JAK2/STAT5 pathway and plays an important role in MPN development. The detailed molecular composition of this signalosome is unclear. Here, we reveal peptidylprolyl isomerase-like 2 (PPIL2) as a critical component of the complex in regulating human and murine erythropoiesis. PPIL2 was a direct target of STAT5 and was upregulated in patients with MPN and in a Jak2^V617F^ MPN mouse model. Mechanistically, PPIL2 interacted with and catalyzed p53 polyubiquitination and proteasome-mediated degradation to promote cell growth. Ppil2 deficiency, or inhibition by cyclosporin A, led to a marked upregulation of p53 in vivo and ameliorated myeloproliferative phenotypes in Jak2^V617F^ mice. Cyclosporin A also markedly reduced JAK2-mutated erythroid and myeloid proliferation in an induced pluripotent stem cell–derived human bone marrow organoid model. Our findings reveal PPIL2 as a critical component of the PLEK2 signalosome in driving MPN pathogenesis through negative regulation of p53, thus providing a target and opportunity for drug repurposing using cyclosporin A to treat MPNs.

## Abstract 2

Our study reveals PPIL2 as one of the major degraders of p53 to drive cell proliferation and provide a rationale to repurpose cyclosporin A for the therapy of myeloproliferative neoplasms.

## Introduction

Myeloproliferative neoplasms (MPNs) are clonal hematopoietic stem cell disorders that are manifested as a progressive expansion of one or more of the myeloid lineages. Studies over the past decade established the central role of the JAK2/STAT pathway in the pathogenesis of MPNs (1–5), which led to the development of several FDA-approved JAK inhibitors for treating various subtypes of MPNs. Although effective in reducing MPN symptoms, JAK inhibitors are not ideal because of their side effects and drug resistance (6–9).

We have been investigating the critical downstream effectors of the JAK2/STAT pathway to identify a drug target that is not only safe to suppress MPNs but also effective. We reported previously that pleckstrin-2 (PLEK2), a membrane phosphoinositide binding protein (10–14), is a major downstream effector of the JAK2/STAT5 pathway. PLEK2 is overexpressed in JAK2^V617F^-positive patients with MPN. Through a mouse genetic approach, we further discovered that the knockout of Plek2 markedly ameliorated the MPN phenotypes of Jak2^V617F^-knockin mice. More importantly, loss of Plek2 markedly reduced the upregulated inflammatory cytokines and thrombosis and markedly extended the survival of Jak2^V617F^-knockin mice (15). This study reveals an essential role of PLEK2 as a critical mediator of the JAK2/STAT5 pathway and suggests PLEK2 as an attractive target for treating MPNs.

More recently, we uncovered the mechanism of PLEK2 by showing that PLEK2 functions as a scaffold protein to directly bind to AKT, which leads to the increased phosphorylation and activation of AKT. Knockout of Plek2 markedly abolished Jak2^V617F^ mutation–induced Akt activation (16). A critical feature of Plek2 knockout mice is that they only show mild anemia at over 1 year of age, which does not affect the overall survival (15). This indicates that the pathophysiologic significance of PLEK2 is mainly exhibited in the JAK2^V617F^ mutant background, which is important for the development of PLEK2 inhibitors to treat MPNs since these agents would be less likely to have severe side effects compared with JAK inhibitors. With this effort, we have developed a class of PLEK2-specific small molecule inhibitors that bind directly to PLEK2 and dissociate the PLEK2-AKT interaction (16). Importantly, these compounds showed potent efficacies in reducing AKT activation and ameliorating MPN phenotypes. No substantial side effects were observed when the compound was used for a long period in mice (16, 17).

These data reveal the essential role of the PLEK2 signalosome complex in the pathogenesis of MPNs and the potential of targeting PLEK2 to treat the disease. They also raise many questions that need to be answered to improve our understanding of the mechanisms of function of the PLEK2 signalosome. Specifically, are there additional components in the complex that play a pivotal role in driving cell proliferation? In this study, we reveal that peptidylprolyl isomerase-like 2 (PPIL2) is a critical component of the PLEK2 signalosome to mediate p53 degradation and drive myeloproliferation. Like PLEK2, PPIL2 is also a target of the JAK2/STAT5 pathway and upregulated in patients with MPNs. These findings reveal a mechanism of p53 suppression in MPNs and provide important therapeutic strategies to elevate p53 in MPNs.

## Results

### PPIL2 is a component of the PLEK2 signalosome and regulates erythropoiesis in vitro.

**Figure 1:**
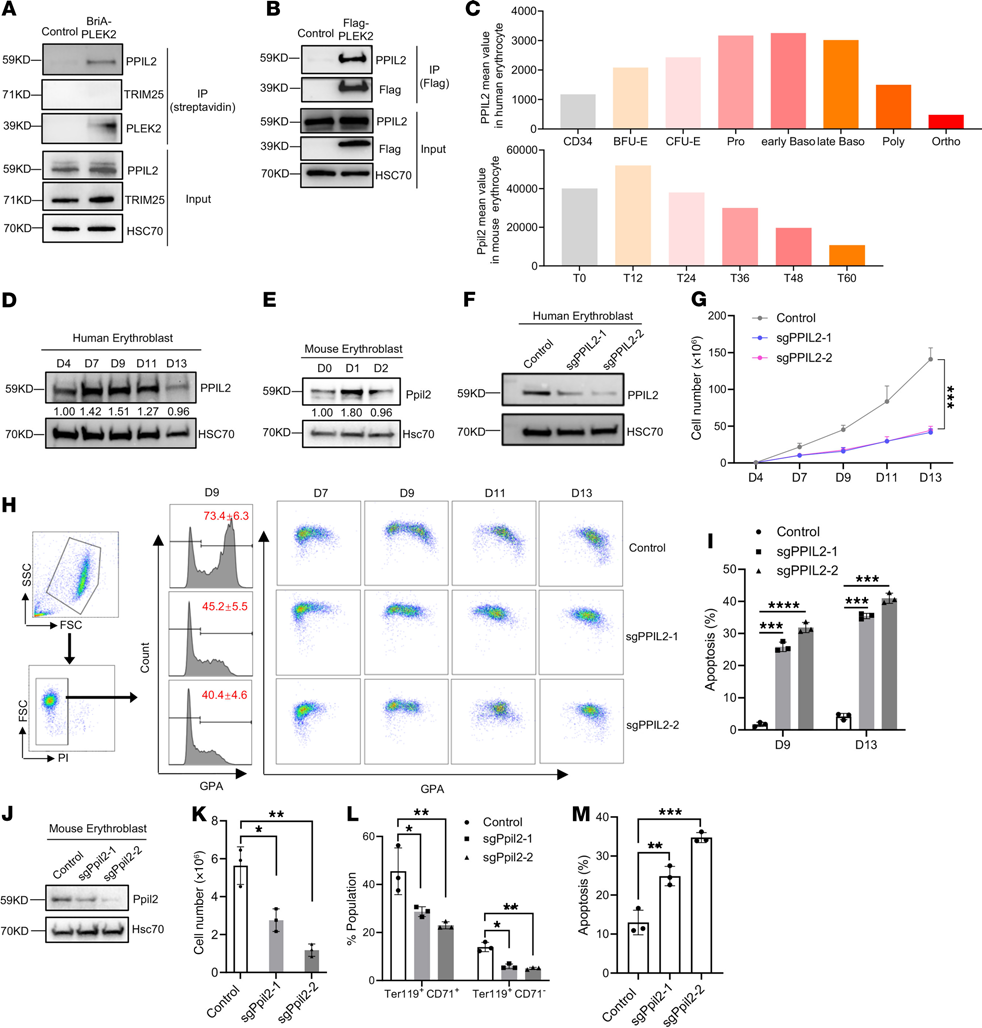
PPIL2 is a component of the PLEK2 signalosome and regulates erythropoiesis in vitro. (**A**) Western blotting of indicated proteins after streptavidin pulldown of lysates from HEK293T cells transfected with control or BirA-PLEK2. (**B**) Western blotting of indicated proteins following anti-Flag IP of lysate from HEK293T cells transfected with control or Flag-PLEK2. (**C**) Relative PPIL2 mRNA levels at the indicated stages of cultured human (upper) and mouse (lower, T: hours) terminal erythroblasts. (**D**) Western blotting of PPIL2 on different days (designated as D) of EPO medium–cultured CD34^+^ cells. HSC70 was used as a loading control. (**E**) Western blotting of Ppil2 at different days of EPO medium–cultured mouse bone marrow lineage-negative cells. (**F**) Western blotting of PPIL2 on day 9 EPO medium–cultured CD34^+^ cells transduced with control or CRISPR-PPIL2 sgRNAs. (**G**) Proliferation assay of cells from **F**. (**H**) Flow cytometric analysis of CD235a (glycophorin A) on day 7 cultured cells from **F**. Representative images of the flow cytometric analyses of CD235a and CD71 on cells of different days are on the right. (**I**) Quantification of cell apoptosis using flow cytometry in cells from **F**. (**J**) Western blotting of Ppil2 in EPO medium–cultured mouse lineage-negative cells transduced with control or CRISPR-Ppil2 sgRNAs. (**K**) Proliferation assays of cells from **J** cultured for 48 hours. (**L**) Quantification of CD71^+^Ter119^+^ erythroid progenitors and CD71-Ter119^+^ mature erythrocytes by flow cytometric assays in cells from **J**. (**M**) Apoptosis assays of cells from **J**. Data are shown as the mean ± SEM. The comparison between 2 groups was evaluated by 2-tailed *t* test (**G**), and the comparison among multiple groups was evaluated with 1-way ANOVA (**I**, **K**, **L**, and **M**). *P < 0.05, **P < 0.01, ***P < 0.001, and ****P < 0.0001.

We previously performed an affinity purification assay using biotin conjugation and streptavidin pulldown of the cell lysate from HEK293T cells expressing BirA-fusion PLEK2 followed by mass spectrometry (16). Besides PLEK2 and the reported HSP72, we found that several E3 ubiquitin ligases, including PPIL2 and TRIM25, were highly enriched in the PLEK2 complex in the mass spectrometry data (16). A repeated streptavidin pulldown assay validated PLEK2 binding to PPIL2 but not TRIM25 (Figure 1A). We further confirmed the interaction between PLEK2 and PPIL2 through a co-IP assay (Figure 1B). PPIL2 is a member of the cyclophilin family of peptidylprolyl isomerase with reported E3 ubiquitin ligase activities and protein folding functions (18, 19). The important role of PLEK2 in erythropoiesis prompted us to study whether PPIL2 is also involved in this process. To this end, we first analyzed the expression levels of PPIL2 in the cultured erythroid cells differentiated from human CD34^+^ hematopoietic stem and progenitor cells (HSPCs). We found an increased PPIL2 expression during terminal erythropoiesis with a decrease in the late stage (Figure 1, C and D). A similar expression pattern was also observed in the cultured mouse bone marrow HSPC-derived erythroid cells (Figure 1, C and E). To test the function, we knocked out PPIL2 in CD34^+^ cells using 2 different PPIL2 sgRNAs, which markedly reduced its level (Figure 1F). PPIL2 deficiency substantially reduced erythroid cell in vitro proliferation (Figure 1G) and differentiation (Figure 1H). There was also a substantial increase in apoptosis (Figure 1I and Supplemental Figure 1, A and C–F; supplemental material available online with this article; https://doi.org/10.1172/JCI181394DS1). The same results were obtained in vitro in the cultured mouse bone marrow HSPC-derived erythroid cells (Figure 1, J–M and Supplemental Figure 1B). In addition, we also found decreased levels of Plek2 and phospho-Akt (Supplemental Figure 1G), indicating that the Plek2 signalosome may have a close functional connection. Overall, these results demonstrate that PPIL2 is important in terminal erythropoiesis in vitro.

### PPIL2 is an E3 ubiquitin ligase of p53.

**Figure 2:**
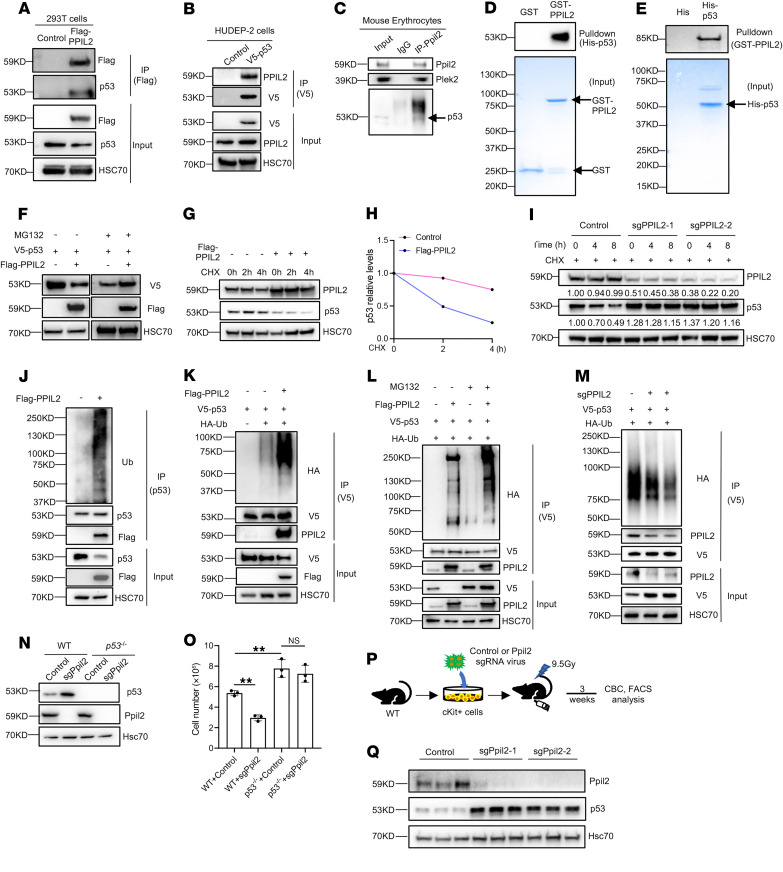
PPIL2 is an E3 ubiquitin ligase of p53. (**A**) Western blotting of indicated proteins following IP of anti-Flag with cell lysate from HEK293T cells transfected with control or Flag-PPIL2. HSC70 was used as a loading control. (**B**) IP of anti-V5 with cell lysate from day 7 cultured human HUDEP-2 cells transduced with V5-p53 followed by Western blotting of indicated proteins. (**C**) Western blotting of indicated proteins following endogenous IP of anti-Ppil2 with cell lysate from mouse bone marrow Ter119^+^ cells. Hsc70 was used as a loading control. (**D**) GST pulldown assays using recombinant GST-PPIL2 with His-p53. (**E**) His-p53 pulldown of GST-tagged PPIL2. (**F**) Western blotting of indicated proteins in HEK293T cells transfected with indicated constructs treated with or without MG132 (1 mM) for 6 hours. (**G**) Western blotting of indicated proteins in HEK293T cells transfected with vector or Flag-PPIL2 treated with or without 2 μg/mL CHX for the indicated time. (**H**) Densitometric analysis of relative p53 protein levels in **G** using ImageJ software. (**I**) Western blotting of indicated proteins in HEK293T cells transiently transduced with control or CRISPR-PPIL2 sgRNA in the presence of 2 μg/mL CHX over indicated time. (**J**) Western blotting of indicated proteins after anti-p53 IP of HEK293T cells transfected with control or Flag-PPIL2. (**K–M**) Western blotting following anti-V5 pulldown from lysates of HEK293T cells transduced with the indicated constructs. (**N**) Western blotting analysis showing expression levels of Ppil2 and p53 in WT or p53 knockout mice bone marrow lineage-negative cells infected with control or CRISPR-Ppil2 sgRNA and cultured in EPO medium. Hsc70 was used as a loading control. (**O**) Proliferation assays of cells from **N** cultured for 48 hours. (**P**) Schematic illustration of experimental design. (**Q**) Western blotting analyses of Ppil2 and p53 expression in the bone marrow of 3-week-old mice transplanted with donor cells transduced with the indicated constructs. Hsc70 was used as a loading control. The comparison among multiple groups was evaluated with 1-way ANOVA (**O**). **P < 0.01.

To explore the mechanism of function of PPIL2 in erythropoiesis, we performed an IP assay using Flag-tagged PPIL2 followed by a Coomassie stain. A prominent band precipitated by Flag-PPIL2 was detected compared with the control group (Supplemental Figure 2A). Mass spectrometry assays revealed known PPIL2 binding proteins, including HSP90 and CRNKL1 (20) (Supplemental Table 1). Among the other interacting proteins, p53 is also enriched. The interaction between PPIL2 and p53 is potentially important, given the role of p53 in cell cycle inhibition and induction of apoptosis. We confirmed PPIL2 and p53 interaction through co-IP assays in HEK293T cells and HUDEP-2 human erythroid cells (Figure 2, A and B). An additional co-IP assay using primary mouse erythroid cells further confirmed the endogenous interaction between Ppil2 and p53, as well as their association with Plek2 (Figure 2C). Interestingly, the p53 protein level was reduced with the ectopic expression of PPIL2, indicating a negative regulatory role of PPIL2 on p53 (Figure 2A). We further performed in vitro pulldown assays using recombinant PPIL2 and p53, demonstrating their direct interactions (Figure 2D–E).

PPIL2 contains a U-box domain linked to its reported E3 ubiquitin ligase activity and a protein-protein interaction (PPI) domain. To map the binding domains between PPIL2 and p53, we constructed GST-tagged PPIL2 mutants with U-box or PPI domains, as well as His-tagged p53 mutants encompassing a single domain or combination of its transcriptional activation domain, DNA-binding domain, and C-terminal regulatory domain. Direct pulldown assays revealed that the U-box domain of PPIL2 and the transcriptional activation domain of p53 are most likely to bind to each other (Supplemental Figure 2, B and C). Notably, the interaction of the p53 transcriptional activation domain to PPIL2 was robust, even though the expression of the p53 transcriptional activation domain was only detectable by Western blotting (Supplemental Figure 2C). These results demonstrate that PPIL2 binds to and downregulates p53, potentially through its E3 ubiquitin ligase activity. This is supported by the finding that PPIL2 overexpression did not affect the transcription of *TP53* (Supplemental Figure 2D). To further demonstrate the inhibitory role of Ppil2 in primary erythroid cells, we used CRISPR/Cas9 to knockout Ppil2 in mouse bone marrow HSPCs (Figure 1K) before culturing them toward terminal erythropoiesis. Indeed, Ppil2 deficiency substantially induced the upregulation of p53 protein but not mRNA levels (Supplemental Figure 2, E and F).

Given the function of PPIL2 as an E3 ubiquitin ligase and in the downregulation of p53, we reasoned that it could mediate p53 polyubiquitination and degradation. To test this, we transiently overexpressed V5-tagged p53 and Flag-tagged PPIL2 in HEK293T cells and treated the cells with a proteasome inhibitor. As expected, overexpression of PPIL2 markedly downregulated p53, which was reverted by the presence of the proteasome inhibitor (Figure 2F). Overexpression of PPIL2 also markedly reduced the half-life of p53 (Figure 2, G and H). Conversely, knockout of PPIL2 increased p53 half-life (Figure 2I). To determine whether PPIL2 directly ubiquitinates p53, we overexpressed Flag-PPIL2 in HEK293T cells and immunoprecipitated p53. Indeed, a Western blotting assay detected polyubiquitination of the precipitated endogenous p53 accompanied by the downregulation of the total p53 level (Figure 2J). Polyubiquitination was also observed in ectopically expressed p53 with overexpression of PPIL2 under normal conditions (Figure 2K) and denatured conditions (Supplemental Figure 2G). Furthermore, when we included the proteasome inhibitor in the system, we found enhanced polyubiquitination and stabilization of p53 (Figure 2L). Consistently, knockout of PPIL2 increased the p53 level and reduced its polyubiquitination (Figure 2M). Next, we analyzed whether the phenotypes of PPIL2 deficiency were dependent on p53. To this end, we knocked out Ppil2 in the bone marrow HSPCs from p53 knockout mice (Figure 2N). When these cells were differentiated into the erythroid lineage in vitro, we did not observe the inhibition of cell proliferation seen in Ppil2-deficient p53 WT cells (Figure 2O). This suggests that the effect of Ppil2 on cell proliferation is, at least in part, p53-dependent. Overall, these data establish PPIL2 as an E3 ligase of p53, leading to its proteasome-mediated degradation.

MDM2 is a well-established E3 ubiquitin ligase of p53. To determine whether the effect of PPIL2 on p53 polyubiquitination and degradation is influenced by MDM2, we knocked down MDM2 in HEK293T cells before ectopically overexpressing PPIL2. We found that p53 was markedly downregulated by PPIL2 with or without the downregulation of MDM2 (Supplemental Figure 2H). This result indicates that the degradation of p53 by PPIL2 is independent of MDM2. Both MDM2 and PPIL2 could be readily precipitated by ectopically expressed p53 (Supplemental Figure 2I). Notably, p53 overexpression markedly upregulated MDM2, a known downstream target, which may explain its increased coprecipitation with p53.

### Loss of Ppil2 in mouse hematopoietic cells in vivo leads to mild anemia.

Given the critical role of PPIL2 in erythropoiesis and p53 downregulation in vitro, we proceeded to study the role of PPIL2 in vivo. Although the *Ppil2* knockout mouse model is unavailable, we took advantage of the bone marrow transplantation approaches using Ppil2-depleted mouse c-Kit^+^ HSPCs (Figure 2P). After 3 weeks, the transplanted mice were analyzed for their complete blood count and bone marrow hematopoiesis. We first confirmed the substantial depletion of Ppil2 in the bone marrow of these mice. Consistent with Ppil2’s E3 enzymatic activity, the p53 level was markedly increased (Figure 2Q). We found mild anemia and neutropenia in mice transplanted with Ppil2-deficient c-Kit^+^ cells. The platelet count remained unchanged (Supplemental Figure 3A). Flow cytometric analyses revealed no significant changes in the bone marrow Ter119^+^ maturing erythroblasts in mice transplanted with Ppil2-deficient c-Kit^+^ cells (Supplemental Figure 3B). However, the erythroid cells were mildly increased in the spleen of these mice (Supplemental Figure 3C), indicating anemia-induced mild stress erythropoiesis. Consistently, the spleen weight in these mice was also mildly increased (Supplemental Figure 3D), and morphologic examination showed mild extramedullary erythropoiesis (Supplemental Figure 3E). To investigate erythropoiesis at different stages of red cell development, we used a well-established CD44-based gating strategy to analyze Ter119^+^ cells (Supplemental Figure 3F). Although there was a slight increase in the percentage of bone marrow early erythroblasts, the number of these cells remained unchanged (Supplemental Figure 3G). The spleen showed an increase in the number of erythroblasts across different stages of terminal differentiation, consistent with mild stress erythropoiesis (Supplemental Figure 3H). Overall, these data reveal that loss of Ppil2 in mouse hematopoietic cells induces a substantial p53 upregulation and a mild hematologic phenotype in vivo.

### PPIL2 is a downstream target of the JAK2/STAT5 pathway and is upregulated in patients with MPNs.

**Figure 3:**
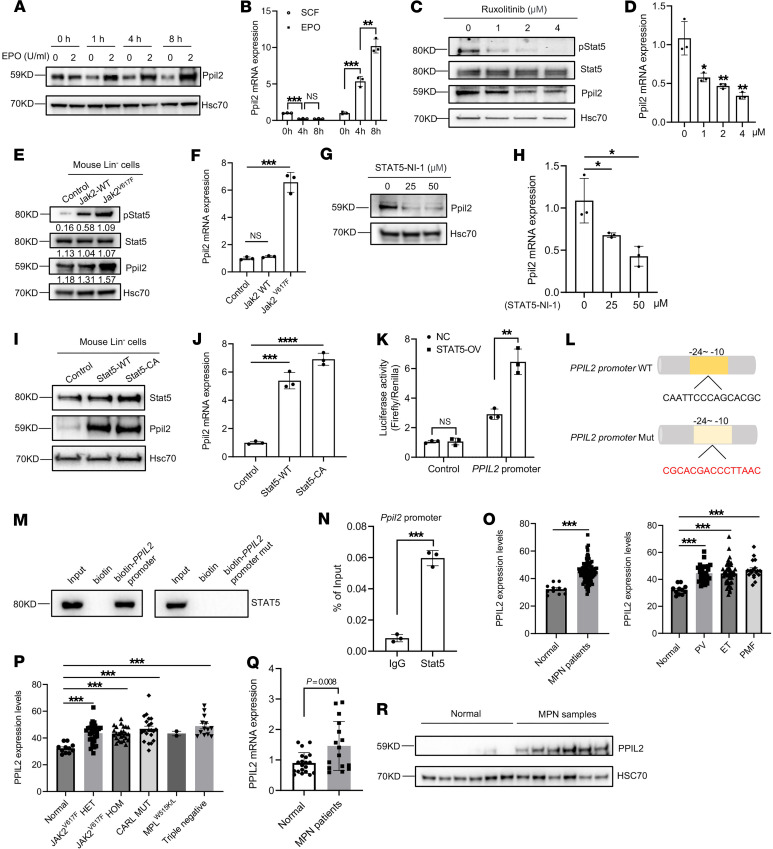
PPIL2 is a downstream target of the JAK2/STAT5 pathway and is upregulated in patients with MPNs. (**A**) Western blotting of Ppil2 in bone marrow lineage-negative cells cultured in SCF medium for 12 hours, then with or without EPO (2 U/mL) for the indicated times. (**B**) qPCR of *Ppil2* mRNA expression in bone marrow lineage-negative cells treated with SCF or EPO over time. (**C** and **D**) Western blot (**C**) and qPCR (**D**) of Ppil2 in cultured erythroblasts treated with varying does of ruxolitinib for 20 hours. Hsc70 was used as a loading control. (**E** and **F**) Western blot (**E**) and qPCR (**F**) of Ppil2 in erythroblasts transduced with WT Jak2 or Jak2^V617F^ mutant. (**G** and **H**) Western blotting (**G**) and PCR (**H**) of Ppil2 in erythroblasts treated with the Stat5 inhibitors for 20 hours. (**I** and **J**) Western blotting (**I**) and qPCR (**J**) of Ppil2 in erythroblasts transduced with WT Stat5 or constitutively active (CA) mutant. (**K**) Relative luciferase activity in HEK293T cells transfected with control or STAT5 (OV) constructs. (**L**) Schematic of STAT5 binding sites on *PPIL2* promoter and mutant sequence. (**M**) DNA pulldown assay by Western blotting using biotin-labeled *PPIL2* promoter or mutant with 24-hour EPO-treated mouse linage-negative cell lysate. (**N**) ChIP-qPCR of Ppil2 promoter fragment pulled down by anti-Stat5 with IgG as the control. (**O** and **P**) *PPIL2* mRNA levels in patients with MPN by subtype (**O**) and mutation status (**P**) from the GSE54644 dataset. (**Q**) *PPIL2* mRNA in PBMCs from an independent cohort of patients with MPN. (**R**) Western blot of PPIL2 in PBMCs from normal and patients with MPN from **Q**. The comparison between 2 groups was evaluated with 2-tailed *t* test (**K**, **N**, **Q**, and **O**), and the comparison among multiple groups was evaluated with 1-way ANOVA (**B**, **D**, **F**, **H**, **J**, **O**, and **P**). ns, nonsignificant; *P < 0.05, **P < 0.01, ***P < 0.001, and **** *P* < 0.0001.

The mild phenotype of Ppil2 hematopoietic deficiency in vivo is similar to Plek2 deficiency. The function of Plek2 as a proto-oncoprotein is mainly manifested when it is overexpressed, which drives Akt activation and cell proliferation (16). Since PPIL2 expression is increased from the CFU-E stage to early terminal erythropoiesis when the cells are sensitive to erythropoietin (EPO) (Figure 1D), we tested whether PPIL2 could also be regulated by the EPO/JAK2/STAT5 pathway. To this end, we purified mouse WT bone marrow lineage-negative cells and cultured the cells in media with or without EPO. EPO treatment led to Ppil2 upregulation within 1 hour, which was sustained over time (Figure 3A). EPO, but not stem cell factor (SCF), also induced upregulation of the *Ppil2* transcript level (Figure 3B). We next treated the cultured mouse erythroblasts with ruxolitinib, a JAK inhibitor, which led to a dose-dependent downregulation of Ppil2 protein (Figure 3C) and transcript levels (Figure 3D). Ruxolitinib treatment also induced the upregulation of caspase-3, consistent with prior studies demonstrating that inhibition of JAK/STAT signaling leads to apoptosis (21, 22) (Supplemental Figure 4A). Notably, the caspase inhibitor failed to prevent the ruxolitinib-induced decrease in PPIL2 protein and mRNA levels (Supplemental Figure 4, B and C), further supporting that ruxolitinib downregulates PPIL2 through the JAK/STAT5 pathway independent of apoptosis. As expected, ectopic expression of Jak2^V617F^, a constitutively active JAK2 mutant, increased the protein and transcript levels of Ppil2 in the cultured erythroid cells (Figure 3, E and F). We also treated the cultured mouse erythroblasts with a STAT5 inhibitor, which showed dose-dependent inhibition of Ppil2 expression in the protein and mRNA levels (Figure 3, G and H). Conversely, ectopic expression of WT or a constitutively active Stat5 increased Ppil2 protein and transcript levels (Figure 3, I and J). To directly test whether Ppil2 is a target gene of STAT5, we performed a luciferase reporter assay, which showed STAT5-induced luciferase activity driven by the *PPIL2* promoter (Figure 3K). In addition, STAT5 bound directly to the biotin-conjugated *PPIL2* promoter, which was abolished when the promoter sequence was mutated (Figure 3, L and M). We also performed a chromatin IP assay followed by quantitative PCR (ChIP-qPCR), which again demonstrated direct interaction of Stat5 with the *Ppil2* promoter region (Figure 3N). Consistent with these data, a previously published report demonstrated STAT5 binding to the *PPIL2* promoter region through a chromatin IP coupled with sequencing (23) (Supplemental Figure 4D). Overall, these data demonstrate that PPIL2 is a direct downstream target of the JAK2/STAT5 pathway.

JAK2^V617F^ mutation is common in patients with MPNs. With the evidence of *PPIL2* as a downstream target of JAK2, we reasoned that PPIL2 could be important in the pathogenesis of MPNs. To test this, we first analyzed *PPIL2* expression using a published database (1). Indeed, *PPIL2* transcription was substantially upregulated in patients with MPN, regardless of the disease subtype (Figure 3O) and mutation status (Figure 3P), which is consistent with the fact that the JAK/STAT pathway is ubiquitously activated in MPNs (1). We next collected PBMCs from an independent cohort of JAK2^V617F^-positive patients with MPN and found the same increase in *PPIL2* transcript levels (Figure 3Q) and, more importantly, in its protein levels (Figure 3R). These data reveal a potentially critical role of PPIL2 in the development of MPNs.

### Ppil2 deficiency ameliorates Jak2^V617F^-induced myeloproliferative phenotypes.

**Figure 4:**
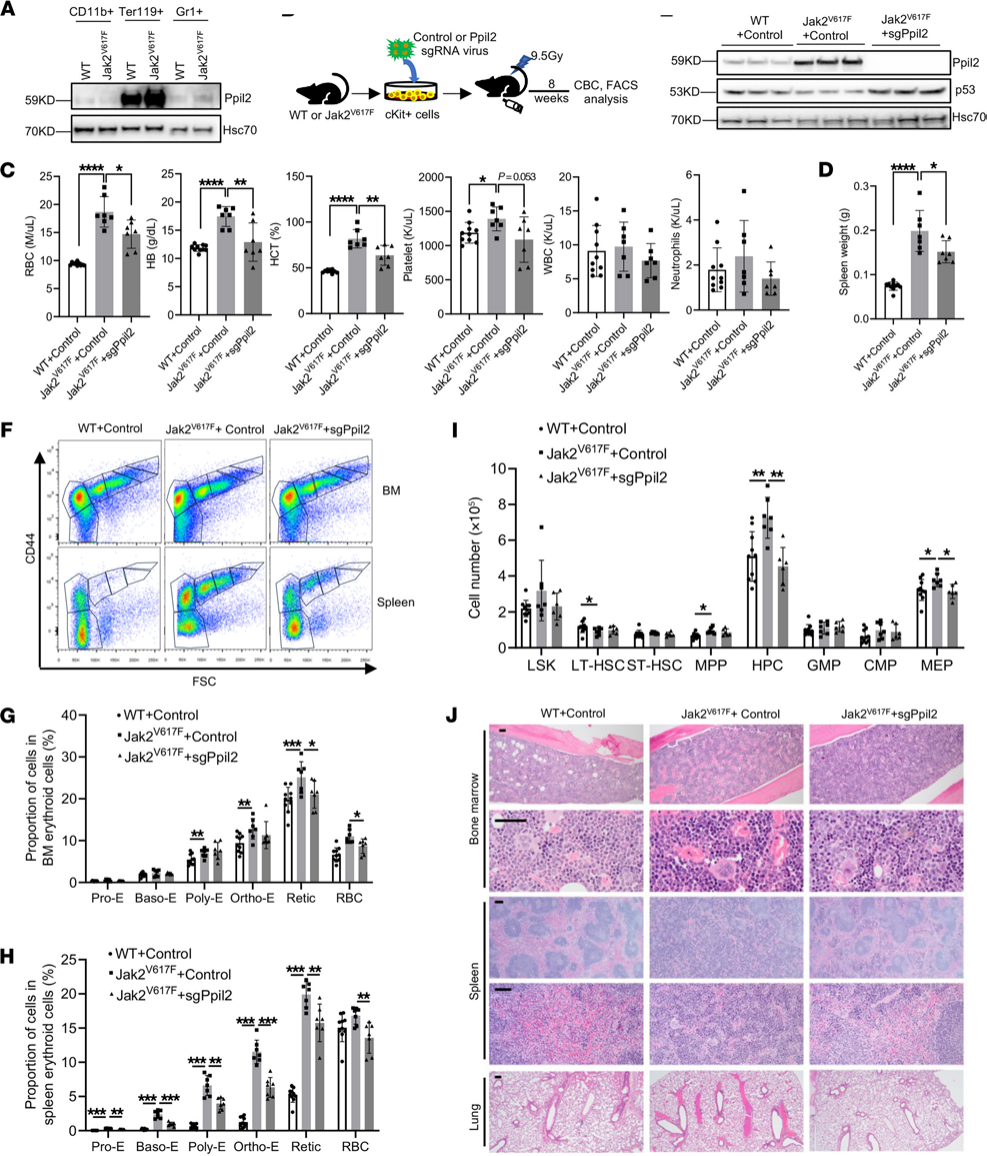
Ppil2 deficiency ameliorates Jak2^V617F^-induced myeloproliferative phenotypes. (**A**) Western blotting of Ppil2 in bone marrow Ter119^+^, CD11b^+^, and Gr1^+^ cells from WT and Jak2^V617F^-knockin mice. Hsc70 was used as a loading control. (**B**) Schematic illustration of experimental design where c-Kit^+^ bone marrow cells from WT or Jak2^V617F^ mice were transduced with control or CRISPR-Ppil2 sgRNA, followed by transplantation into lethally irradiated recipient mice. The recipient mice were examined for hematologic phenotypes 8 weeks after transplant. (**C**) Complete blood count of mice from **B**. WT+Control: *n* = 10, Jak2^V617F^+Control: *n* = 7, Jak2^V617F^+sgPpil2: *n* = 7. (**D**) Quantification of spleen weight of mice from **B**. (**E**) Western blotting of Ppil2 and p53 expression in the bone marrow of indicated mice in **B**. (**F**) Representative flow cytometric analyses of terminal erythropoiesis using CD44 and forward scatter as markers. Populations I-VI represent proerythroblasts, basophilic erythroblasts, polychromatic erythroblasts, orthochromatic erythroblasts, reticulocytes, and mature RBCs, respectively. (**G** and **H**) Quantification of different stages of erythroblasts by flow cytometric analyses as **F** from bone marrow (**G**) and spleen (**H**). (**I**) Quantification of HSPCs by flow cytometric analyses from the bone marrow in mice from **B**. HPC: lineage negative, c-Kit^+^ hematopoietic progenitor cells. GMP, granulocyte-macrophage progenitor; CMP, common myeloid progenitor; MEP, megakaryocyte-erythrocyte progenitor; LSK, Lin^–^Sca1^+^cKit^+^ cells; LT-HSC, long-term HSC; ST-HSC, short-term HSC; MPP, multipotential progenitor. (**J**) Representative H&E staining of the indicated organs of the indicated mice from **B**. Scale bars: 100 μm. The comparison among multiple groups was evaluated with 1-way ANOVA (**C**, **D**, **I**, **G**, and **H**). *P < 0.05, **P < 0.01, ***P < 0.001, and ****P < 0.0001.

To further investigate the role of PPIL2 in MPNs in vivo, we utilized a Jak2^V617F^-knockin mouse model that closely resembles patients with MPNs (24). Ppil2 is highly expressed in the erythroid lineage in these mice (Figure 4A), which is consistent with its role in erythropoiesis. We purified the bone marrow c-Kit^+^ HSPCs from Jak2^V617F^-knockin or WT mice and transduced them with *Ppil2* sgRNA and Cas9. These cells were then transplanted into lethally irradiated recipient mice. These mice were analyzed 8 weeks after transplantation for MPN phenotypes (Figure 4B). We found a significant reduction in RBC count, hemoglobin, hematocrit, and a reduction trend in platelet and neutrophil counts in mice transplanted with Ppil2-deficient c-Kit^+^ cells (Figure 4C). The spleen weight was also markedly reduced (Figure 4D). Western blotting assays of the total bone marrow cells confirmed the upregulation of Ppil2 in Jak2^V617F^ mice and efficient depletion of the gene by the sgRNA. More importantly, the level of p53, which was expectedly reduced in Jak2^V617F^ cells, was markedly upregulated with Ppil2 deficiency (Figure 4E).

We euthanized these mice and purified their bone marrow and spleen cells. Mice transplanted with Jak2^V617F^ c-Kit^+^ cells showed marked upregulation of erythroblasts across all the developmental stages during terminal erythropoiesis in both bone marrow and spleen. This erythroid hyperplasia, especially in the spleen, was substantially ameliorated with the loss of Ppil2 (Figure 4, F–H). In the HSPC populations, Jak2^V617F^ induced upregulation mainly in the megakaryocyte-erythrocyte progenitors and c-Kit^+^ HPC population, which was reverted with Ppil2 deficiency (Figure 4I and Supplemental Figure 4E). Morphologically, Jak2^V617F^ transplanted mice showed increased bone marrow cellularity, extramedullary erythropoiesis, and marked pulmonary thrombosis, which were markedly reverted by the loss of Ppil2 (Figure 4J). Overall, these results demonstrate that Ppil2 is important in mediating the downregulation of p53 in vivo and promoting MPN pathogenesis.

### Inhibition of Ppil2 blocks erythropoiesis in vitro and ameliorates MPN symptoms in Jak2^V617F^-knockin mice.

**Figure 5:**
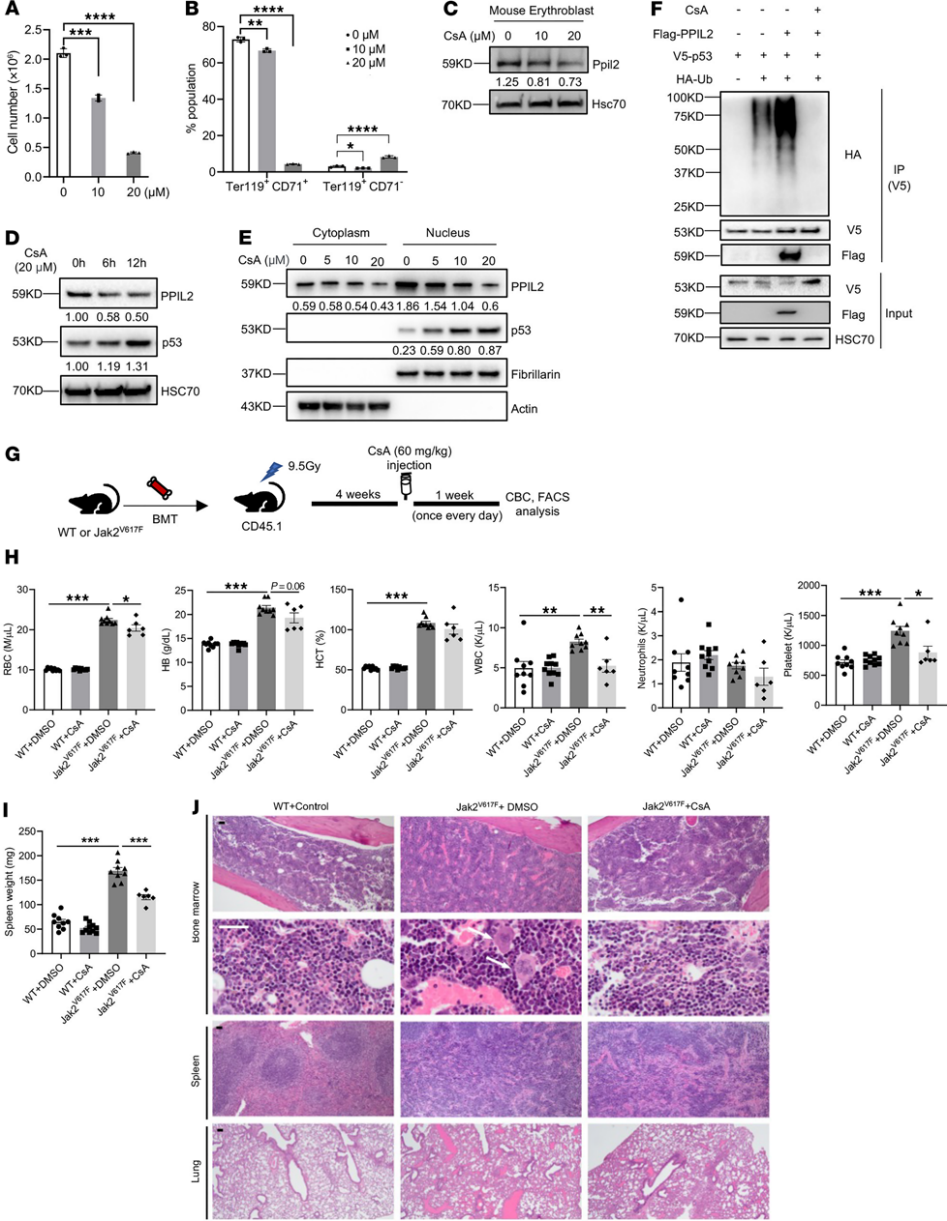
Inhibition of Ppil2 blocks erythropoiesis in vitro and ameliorates MPN symptoms in Jak2^V617F^-knockin mice. (**A**) Mouse bone marrow lineage-negative cells were cultured in EPO medium for 2 days in the presence of the indicated amount of cyclosporin A (CsA). The cell number was quantified on day 2. (**B**) Quantification of indicated cell populations detected by flow cytometry in cells from **A**. (**C**) Western blotting of Ppil2 in cells from **A**. Hsc70 was used as a loading control. (**D**) Western blotting of the indicated proteins in HEK293T cells treated with 20 μM CsA for the indicated amount of time. (**E**) Western blotting of indicated proteins in the nuclear and cytoplasmic fractions of HEK293T cells treated with the indicated amount of CsA for 12 hours. (**F**) Western blotting of indicated proteins following anti-V5 IP of HEK293T cells transfected with the indicated constructs and treated with or without 20 μM CsA for 12 hours before being harvested. (**G**) Schematic illustration of experimental design where total bone marrow cells from 2-month-old Jak2^V617F^-knockin mice (CD45.2^+^) were transplanted into lethally irradiated recipient mice (CD45.1^+^). One month after transplant, the recipient mice were treated with 60 mg/kg CsA or vehicle control once every day for 1 week. (**H**) Complete blood count of the indicated mice from **G**. Control: *n* = 9, CsA: *n* = 10, Jak2^V617F^: *n* = 9, Jak2^V617F^+CsA: *n* = 6. (**I**) Spleen weight of the indicated mice from **G**. (**J**) Representative H&E staining of the indicated organs from the indicated mice in **G**. Scale bars: 100 μm. Arrows point to enlarged megakaryocytes in Jak2^V617F^ mice. The comparison among multiple groups was evaluated with 1-way ANOVA (**A**, **B**, **I**, and **H**). *P < 0.05, **P < 0.01, ***P < 0.001, and ****P < 0.0001.

The U-box ubiquitin E3 ligases are major cytosolic binding proteins of cyclosporin A (CsA), an immunosuppressive drug targeting the cyclophilin family of peptidylprolyl isomerases, including PPIL2 (25–28). To determine whether CsA also functionally inhibits PPIL2 in erythropoiesis, we treated cultured mouse bone marrow erythroblasts with CsA, which led to a dose-dependent inhibition of cell proliferation and differentiation (Figure 5, A and B). The protein level of Ppil2 was also reduced (Figure 5C). As expected, p53 was upregulated with the reduction and inhibition of PPIL2 (Figure 5D). This effect of CsA on the level of PPIL2 and p53 was dose-dependent and mainly occurred in the nucleus, although PPIL2 is located both in the cytoplasm and nucleus (Figure 5E). Consistent with the mechanism of PPIL2, CsA treatment abolished the ubiquitination and degradation of p53 (Figure 5F).

To determine the potential therapeutic effects of CsA in MPNs, we transplanted the bone marrow cells from Jak2^V617F^ mice into lethally irradiated recipient mice, which developed MPNs 1 month after transplant. We then treated these mice with 60 mg/kg CsA through daily intraperitoneal injections for 1 week (Figure 5G). Indeed, CsA treatment markedly reduced RBC, WBC, and platelet counts (Figure 5H). The spleen weight was also markedly reduced (Figure 5I). As expected, CsA treatment also reduced bone marrow cellularity, megakaryocytic atypia, splenic extramedullary erythropoiesis, and pulmonary thrombosis (Figure 5J).

### CsA combined with MDM2 inhibitors show enhanced efficacies in ameliorating MPNs in vivo.

The upregulation of WT p53 is an important strategy for treating MPNs. In this respect, small molecule inhibitors of MDM2, including nutlin and its derivative RG7112, have been studied in MPN models and clinical trials and have shown their efficacies (29, 30). To determine whether CsA in combination with MDM2 inhibitors improves the efficacies in treating MPNs, we first performed an in vitro assay in which mouse bone marrow HSPCs cultured in EPO medium were treated with CsA, nutlin, RG7112, or CsA combined with nutlin or RG7112. Indeed, the combined treatments were more effective than single compounds in reducing cell proliferation (Supplemental Figure 5A), blocking cell differentiation (Supplemental Figure 5B), and inducing cell death (Supplemental Figure 5, C and D).

**Figure 6:**
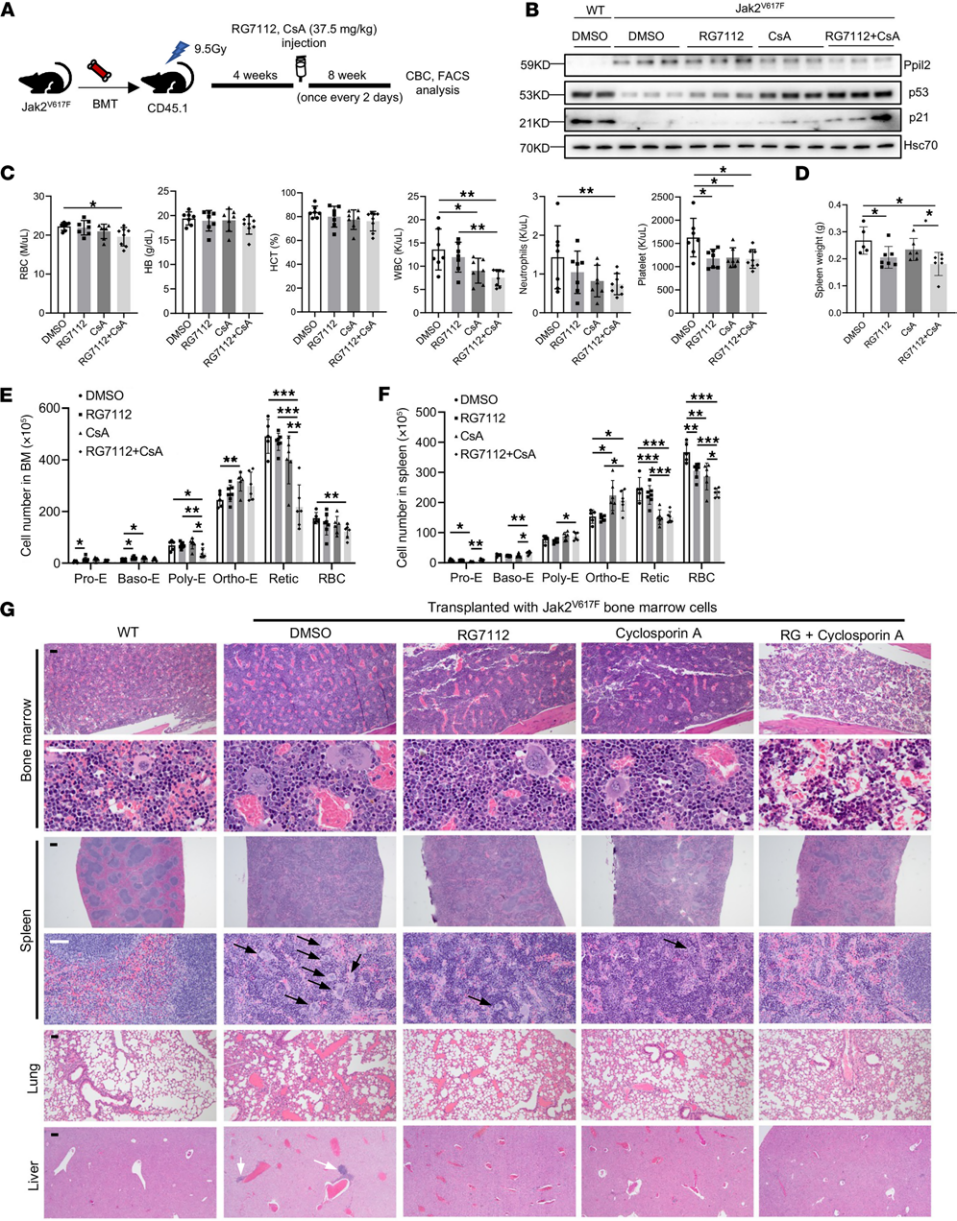
Cyclosporin A and MDM2 inhibitors show enhanced efficacies in ameliorating MPNs in vivo. (**A**) Schematic illustration of experimental design where total bone marrow cells from 2-month-old Jak2^V617F^-knockin mice (CD45.2^+^) were transplanted into lethally irradiated recipient mice (CD45.1^+^). One month after transplant, the recipient mice were treated with CsA or RG7112 alone or in combination once every 2 days for 8 weeks. (**B**) Western blotting analyses of indicated proteins in the spleen of indicated mice in **A**. Hsc70 was used as a loading control. (**C**) Complete blood count of the mice from **A**. Control: *n* = 5, RG7112: *n* = 7, CsA: *n* = 6, RG7112 + CsA: *n* = 6. (**D**) Quantification of spleen weight in each group in **A**. (**E** and **F**) Quantification of different stages of erythroblasts by flow cytometric analyses from the bone marrow (**E**) and spleen (**F**) in mice from **A**. (**G**) Representative H&E staining of the indicated organs from the indicated mice in **A**. Arrows indicate megakaryocytes. Scale bar: 100 μm. The comparison among multiple groups was evaluated with 1-way ANOVA (**C**–**F**). *P < 0.05, **P < 0.01, ***P < 0.001.

We then combined CsA with nutlin derivative RG7112 to study the in vivo effects. We took the same approach as in the CsA single regimen in Figure 5, except with reduced CsA dose and prolonged treatment (Figure 6A). We found that with the reduced dose, CsA alone remained effective in reducing Ppil2 and upregulating p53 and p21 (Figure 6B). Combined therapy also reduced RBC, WBC, neutrophil, and platelet counts, which remained elevated with single compound low-dose therapy (Figure 6C). The spleen weight was also reduced with the combined therapy (Figure 6D). In the bone marrow and spleen, combination therapy markedly reduced terminal erythropoiesis mainly in the late stage of terminal erythroblasts, including late-stage orthochromatic erythroblasts, reticulocytes, and mature RBCs (Figure 6, E and F). Morphologically, combined therapy markedly reduced bone marrow cellularity and atypical megakaryocytes. The spleen’s normal architecture that was disrupted by the extramedullary hematopoiesis was also partially recovered with combined treatment. The combined treatment also markedly reduced pulmonary and liver thrombosis (Figure 6G).

CsA has multiple targets and pleiotropic functions. To determine whether PPIL2 is a major mediator of CsA, we knocked out Ppil2 through CRISPR/Cas9 in the lineage-negative HSPCs from the JAK2^V617F^ knockin mice and treated them with CsA in vitro in the EPO culture medium. We found no marked difference between CsA-treated Ppil2-knockout JAK2^V617F^ cells and their DMSO-treated counterparts (Supplemental Figure 5, E and F). These results suggest that CsA functions mainly through Ppil2 in the JAK2^V617F^-positive MPNs.

### JAK2^V617F^-mutated MPN patient bone marrow cells engrafted in a bone marrow organoid model are more sensitive to CsA treatment.

**Figure 7:**
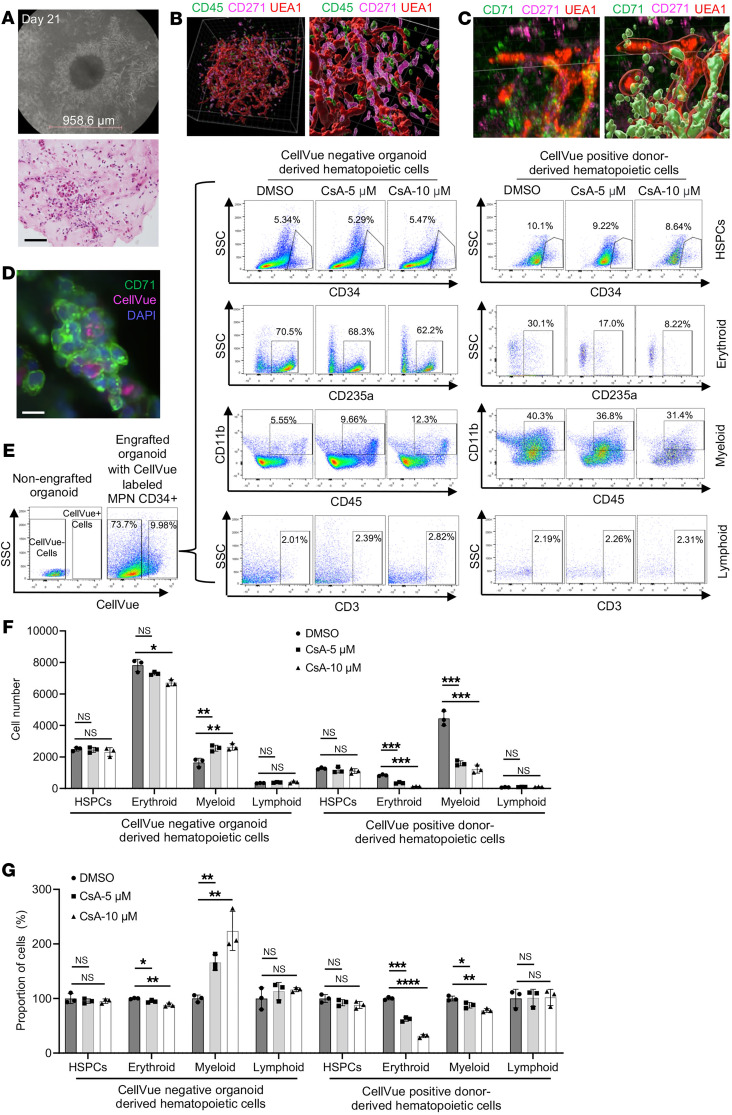
JAK2^V617F^-mutated MPN patient bone marrow cells engrafted in a bone marrow organoid model are more sensitive to CsA treatment. (**A**) Representative wide-field picture (top) and H&E stain (bottom) of day 21 iPSC-derived bone marrow organoid in culture. Scale bar: 100 μm. (**B** and **C**) Whole-mount 3D imaging of the organoids. Imaris was used for cell surface rendering on the right panels. Organoids were stained with indicated antibodies and subsequently imaged in *Z*-stack on Nikon ARX laser scanning confocal platform. (**D**) CellVue-labeled donor CD34^+^ HSPCs (5,000) from a JAK2^V617F^-positive patient with MPN were coincubated with an iPSC-derived bone marrow organoid for 3 days in each well of a 96-well plate, followed by an immunofluorescence assay. Representative pictures show the engraftment of donor hematopoietic cells into the organoid. (**E**) Flow cytometry of the organoids using indicated antibodies for various lineages of the organoid-derived (CellVue negative) and engrafted JAK2^V617F^-positive donor cells (CellVue positive) from **D**. In each group, 20 organoids were collected for the assay. (**F**) Quantitative analyses of the flow cytometry assays in **E**. (**G**) Same as **F** except that the DMSO groups are normalized to 100%. The comparison among multiple groups was evaluated with 1-way ANOVA (**F** and **G**). *P < 0.05, **P < 0.01, ***P < 0.001.

To extend our findings to the human bone marrow context, we applied an induced pluripotent stem cell–derived (iPSC-derived) human bone marrow organoid system that we recently developed (31). These organoids closely resemble human bone marrow biopsy specimens (Figure 7A), similar to what has been reported (32, 33). Whole-mount 3D imaging revealed an endothelial network with stromal cells covering the capillary wall and hematopoietic cells arranged in clusters within and outside the vessels (Figure 7, B and C). We previously showed that these organoids can be applied to study human ex vivo bone marrow engraftment using patient-derived CD34^+^ cells (31). Therefore, we incubated the organoids with CellVue-labeled donor bone marrow CD34^+^ cells from a patient with JAK2^V617F^-positive MPNs for 3 days. The donor cells could be readily detected and surrounded by the recipient hematopoietic cells in the organoids (Figure 7D). We then performed flow cytometry assays of CellVue-positive donor and CellVue-negative recipient populations from the engrafted organoids. We found that multilineage hematopoiesis could be readily detected in both populations (Figure 7E). Notably, the percentage of the erythroid population was relatively high in the CellVue-negative organoid recipient cells, possibly due to the accumulation of enucleated reticulocytes and mature RBCs (Figure 7E). Consistent with our findings in vitro and in the mouse models, CsA treatment led to dose-dependent reductions of the engrafted erythroid and myeloid populations from the donor JAK2^V617F^-positive patients. There was also a minor decrease in the CellVue-negative organoid-derived erythroid cells. However, the extent of the reduction, which reflects their sensitivities to CsA, was far less than that of the donor JAK2^V617F^-positive cells (Figure 7, F and G).

## Discussion

In this study, we revealed a critical role of PPIL2 as a downstream target of the JAK2/STAT5 pathway in mediating the ubiquitination and degradation of p53 to promote myeloproliferation. PPIL2 is a member of the cyclophilin family of peptidylprolyl isomerases and is reportedly functional in protein folding (19). Contradictory studies showed that PPIL2 functions as an oncoprotein or a tumor suppressor in different cancers (18, 34, 35), indicating its tissue-specific roles. Recent structural studies also identified PPIL2 as a critical component of the minor spliceosome (36). Through affinity binding assays coupled with proteomic studies, we found that PPIL2 is a critical component of the PLEK2 signalosome. The other important proteins in this complex include AKT and its effector proteins. Once PLEK2 is overexpressed by the activated JAK2/STAT5 pathway, it functions as a scaffold protein to recruit and activate AKT to promote cell proliferation. The identification of PPIL2 in the complex uncovers that PLEK2 signalosome promotes cell proliferation not only by activating oncoprotein AKT but also by degrading tumor suppressor p53.

In addition to PPIL2, many E3 ubiquitin ligases of p53 have been identified. Among these, the most well-studied is MDM2. Mdm2 deficiency in mice induces embryonic lethality, which can be rescued by the absence of p53 (37). It is unclear whether Ppil2 constitutive deletion leads to embryonic lethality. In this respect, the CRISPR/Cas9 knockout experiment in HSPCs followed by transplantation revealed mild anemia and cytopenia in the recipient mice, confirming Ppil2’s function in hematopoiesis. The knockout efficacies in this system were high with minimally detectable Ppil2 protein. These results indicate that Ppil2 is not essential for hematopoiesis under physiologic conditions. Notably, the p53 level was markedly upregulated in the bone marrow of these mice. The mild hematologic phenotype is consistent with previous studies showing that an increase in p53 activity does not necessarily result in severe defects in mice (38–42). These findings also support the candidacy of PPIL2 as a target for MPN therapy since patients will have fewer side effects when PPIL2’s function is compromised. The same as PLEK2, PPIL2 plays an essential role in driving cell proliferation only when the protein is overexpressed. The definitive in vivo role of Ppil2 in p53 hemostasis needs the generation of a Ppil2 knockout mouse model. Nevertheless, the robust p53 elevation after CsA treatment or PPIL2 knockdown in vitro points to a critical role of PPIL2 in regulating p53-mediated antiproliferative effects, especially in the hyperproliferative condition when JAK2 is mutated.

The potential role of PPIL2 in regulating the p53 level was further indicated by previous studies showing that CsA upregulates the expression of p53 and p21 (43). We found that CsA reduced the expression of PPIL2, which could be the mechanism that leads to the upregulation of p53. It is unclear how CsA reduces the level of PPIL2. It is also possible that CsA inhibits PPIL2’s peptidyl-prolyl isomerase (PPIase) activity, which is essential for its function. CsA could also disrupt PPIL2-p53 binding, either by blocking the binding site or altering PPIL2’s conformation. As a broad inhibitor of the cyclophilin family of peptidylprolyl isomerases, CsA could function nonspecifically and indirectly decrease the PPIL2 level. Although future efforts to develop specific PPIL2 inhibitors could be beneficial, our study shows that CsA is a potent drug that can be used singly or combined with the current therapeutic approaches in MPNs. In particular, CsA was more effective in upregulating p53 and its downstream targets than MDM2 inhibitor RG7112 in our in vivo animal model. Treatment of the Jak2^V617F^ mouse model with CsA alone markedly ameliorated MPN phenotypes with better efficacies than RG7112 single treatment, although their direct comparison requires further pharmacokinetic studies. Notably, the combination of CsA with RG7112 showed improved p53 upregulation and efficacies in reducing MPN phenotypes in Jak2^V617F^ mice. These findings strongly suggest that CsA could be repurposed to treat MPNs. In this respect, CsA has been used to treat myelodysplastic syndromes as an off-label approach (44, 45), which is presumed to work through immunosuppression. It is likely that p53 upregulation also plays an important role in this setting.

These findings in the murine models were further confirmed in the human setting through an iPSC-derived human bone marrow organoid model. The organoids provide a bone marrow microenvironment that supports the hematopoiesis of the engrafted patient-derived HSPCs. When fluorescently labeled CD34^+^ HSPCs were coincubated with the organoid, they migrated toward the vasculature niche and initiated multilineage hematopoietic differentiation within the organoid environment, as confirmed by flow cytometry assays. These data underscore the capability of our human bone marrow organoid model to accurately simulate and sustain the human bone marrow microenvironment.

## Methods

### Sex as a biological variable.

Mice of both sexes were included unless otherwise stated. No substantial differences were observed between sexes for the primary phenotype, and therefore, the data from both sexes were combined for presentation. All observers were blinded to the experimental conditions.

### Mice.

C57BL/6 mice were purchased from the Jackson Laboratory (strain 000664). *p53* knockout mice (C57BL/6 background) were provided by Yan Liu (Northwestern University, Chicago, Illinois, USA). Jak2^V617F^ floxed mice (C57BL/6 background) were provided by Benjamin Ebert and Ann Mullally (Harvard Medical School, Boston, Massachusetts, USA). For the in vivo assays of CsA in Jak2^V617F^-knockin mice, total bone marrow cells from Jak2^V617F^-knockin mice (CD45.2^+^) were transplanted into lethally irradiated recipient mice (CD45.1^+^). After 4 weeks, the recipient mice were treated with 60 mg/kg CsA or vehicle control daily for 1 week. For Jak2^V617F^-knockin mice treated with CsA and RG7112 (Selleck), the transplant recipient mice were treated with 20 mg/kg CsA and 20 mg/kg RG7112 every 2 days for 1 week, followed by 37.5 mg/kg CsA and 37.5 mg/kg RG7112 every 2 days for 7 weeks, or vehicle control every 2 days for 8 weeks. All the treatments were performed by intraperitoneal injection. For the analyses of complete blood counts, peripheral blood (70 μL from each mouse) was collected from the retro-orbital vein in EDTA-coated tubes and analyzed by a Hemavet 950 complete blood counter (Drew Scientific). For the pathologic examination, mice were euthanized with CO_2_, and organs were dissected. Specifically, bone marrow, spleen, liver, and lungs were collected and fixed in 10% neutral buffered formalin and embedded in paraffin for histological and pathological examination. All animal studies followed the *Guide for the Care and Use of Laboratory Animals* (National Academies Press, 2011) and were approved by the IACUC at Northwestern University.

### Cell culture.

Purification of mouse lineage-negative and in vitro culture were performed as previously described (16, 46–49). Briefly, for erythroblast differentiation, purified lineage-negative cells were cultured in IMDM containing 15% FBS (StemCell Technologies), 1% detoxified BSA (StemCell Technologies), 200 μg/mL holo-transferrin (MilliporeSigma), 10 μg/mL recombinant human insulin (MilliporeSigma), 2 mM L-glutamine, 10^–4^ M β-mercaptoethanol, and 2 U/mL recombinant human EPO (Amgen). The cells were then cultured for 2 days before being collected for the subsequent experiments. The procedure of human CD34^+^ cells (StemCell Technologies) differentiated to erythropoiesis had 3 phases. The cells were first cultured in IMDM containing 2% human AB plasma, 3% human AB serum, 200 μg/mL human holo-transferrin, 3 IU/mL heparin, 10 μg/mL insulin, and 1% penicillin/streptomycin. In phase I (days 0–6), CD34^+^ cells at a concentration of 10^5^/mL were supplemented with 10 ng/mL SCF, 1 ng/mL IL-3, and 3 IU/mL EPO. In phase II (days 7–11), the cells were supplemented with 1 IU/mL EPO and 10 ng/mL SCF alone. In phase III (days 12–21), the cell concentration was adjusted to 1 × 10^6^/mL on day 11 and 5 × 10^6^/mL on day 15. The medium for this phase was the base medium containing 1 IU/mL EPO and 1 mg/mL holo-transferrin. The cells were split into fresh culture medium every 3 days. For HEK293T cells (ATCC), the cells were cultured in DMEM supplemented with 10% FBS (Gibco). The cells were cultured at 37°C in 5% CO_2_.

HUDEP-2 cells were obtained from the Riken Institute and were cultured as described (50). Briefly, cells were maintained in media containing StemSpan SFEM (StemCell Technologies) supplemented with 50 ng/mL human SCF, 3 IU/mL EPO, 0.4 μg/mL dexamethasone (Sigma-Aldrich), and 1 μg/mL doxycycline (Sigma-Aldrich). Cell densities throughout the culture were kept under 0.8 × 10^6^/mL. Erythroid differentiation was induced by placing 2 × 10^6^ to 5 × 10^6^ HUDEP-2 cells into IMDM supplemented with 1% L-glutamine, 330 μg/mL human holo-transferrin, 10 μg/mL insulin, 2 IU/mL heparin, 5% FBS, 3 IU/mL EPO, and 1 μg/mL doxycycline. Fresh media were replaced on day 3 and maintained for another 2–3 days before cells were used for analysis.

### Plasmids and transfection.

pLenti-V5-p53 (plasmid 22945), pET15b-p53 (plasmid 24859), lentiCRISPR v2 (plasmid 52961), MAC-STA5A (plasmid 167799) were purchased from Addgene. pGL-4.53 (plasmid E6681) was purchased from Promega. pAX2, pMD2G, Pcl-Eco, HA-Ub, MSCV-IRES-GFP-Stat5 WT; dominant-negative, and constitutively active mutant plasmids, MSCV-IRES-GFP-Jak2 WT, and V617F mutant plasmids were previously reported (15, 16). pCMV-Flag-PPIL2, pNL1.1-*PPIL2* and mutant promoter plasmids, prokaryotic expression vectors pET15b-His-p53 (1-97), 98-292, 293-393, pGEX6p-3-GST-PPIL2 (1-520), 1-190, and 275-435 were individually cloned. For plasmid transfection, HEK293T cells were transfected using TransIT-LT1 transfection reagent (Mirus) according to the manufacturer’s protocol.

### Virus packaging and infection.

For human and mouse CRISPR-PPIL2 sgRNA and human CRISPR-MDM2 sgRNA, oligonucleotides were cloned into the lentiviral vector lentiCRISPR v2 (Addgene) containing the Cas9 gene according to the manufacturer’s protocol. Lentiviruses were packaged in HEK293T cells by transfection with the plasmids mentioned above and with the lentiviral packaging constructs pAX2 and pMD2G in a 4:3:1 ratio by TransIT-LT1 transfection reagent (Mirus) according to the manufacturer’s protocol. Retroviral constructs MSCV-IRES-GFP-Stat5 WT and constitutively active mutants were provided by Merav Socolovsky (University of Massachusetts, Worcester, MA, USA). MSCV-IRES-GFP-Jak2 WT and V617F mutants were provided by Lily Huang (University of Texas Southwestern Medical Center, Dallas, TX, USA). Retroviruses were packaged in HEK293T cells by transfection with the plasmids mentioned above and with the retroviral packaging constructs Pcl-Eco in a 2:1 ratio by TransIT-LT1 transfection reagent according to the manufacturer’s protocol (Mirus). For viral infection, 30 million control, human ROSA26 sgRNA, or human PPIL2 sgRNA lentiviral particles were used to infect 0.5 million human CD34^+^ cells induced to erythroid cells on day 2 with 8 μg/mL polybrene (Millipore Sigma). After 14 hours of infection, cells were washed with PBS and cultured in a new complete medium for 48 hours (day 4). Cells were then selected with 1 μg/mL puromycin until the end of the culture period. Starting at day 7, the extent of terminal erythroid cells was monitored by flow cytometry. For the infection of mouse bone marrow HSPCs, 30 million retroviruses were used to infect 0.5 million bone marrow lineage-negative cells with 8 μg/mL polybrene and centrifuged at 500 *g*. for 1 hour at 37°C. After spin-infection, the viral supernatants were immediately removed, and fresh media were added. To infect HEK293T cells, 10 million control, human PPIL2 sgRNA, or MDM2 sgRNA retroviral particles were used to infect 1 million HEK293T cells with 8 μg/mL polybrene. After 12 hours of infection, cells were washed with PBS and cultured in a new complete medium for 48 hours.

### Flow cytometric assays.

For human terminal erythroid cells, 2 × 105 cells were collected every other day from day 7 to day 13 of culture and prepared by resuspending the cells in PBS with 0.5% BSA (Santa Cruz Biotechnology) and 2 mM EDTA (Gibco). Cells were then stained with CD235a-PE-Cy7 (BioLegend, 349112) and CD71-FITC (eBioscience, 2460053). Propidium iodide was used as a viability marker. For mouse HSPCs cultured in EPO medium for erythroid differentiation, 2 × 10^5^ cells were suspended in PBS with 0.5% BSA and 2 mM EDTA and stained with Ter119-APC (eBioscience,17-5921-83), CD71-FITC (eBioscience,11-0711-85), Hoechst-33342-BV421 (Thermo Fisher Scientific, H3570), and propidium iodide. For primary mouse terminal erythroid cells, single-cell suspensions of bone marrow and spleen were prepared by resuspending the cells in PBS with 0.5% BSA and 2 mM EDTA. Cells were stained with the following antibodies: Ter119-APC (eBioscience, 17-5921-83), CD44-FITC (BioLegend, 103006), CD45-PE (eBioscience, 12-0454-83), Gr1-PE (eBioscience, 12-5931-83), and CD11b-PE (eBioscience, 12-0112-83). For mouse HSPC characterization, lineage-negative cells were purified from bone marrow and resuspended in PBS with 0.5% BSA and 2 mM EDTA. Cells were stained with the following antibodies: Sca1-FITC (BioLegend, 160908), cKit-BV421 (BioLegend, 105820), CD34-PE (BioLegend, 128610), CD135-APC (BioLegend, 135310), and CD16/32 (BioLegend, 156608). All staining was performed for 30 minutes at room temperature. The samples were analyzed using a FACSCalibur flow cytometer (BD Biosciences). Post-acquisition analyses were performed with FlowJo software v9.2.3 (Tree Star).

### Real-time qPCR and Western blotting.

Total cellular RNA was purified using TRIzol (Invitrogen). Next, 1 μg of total RNA was used to synthesize cDNA using Superscript III (Invitrogen), as described by the manufacturer. RT-qPCR was performed using PerfeCTa SYBR Green qPCR FastMix ROX (Quanta BioSciences, Inc.) in an ABI StepOnePlus instrument (Applied Biosystems). Relative gene expression was calculated using the 2^–ΔΔCT^ method, with 18s as a reference gene.

For Western blotting analysis, cells were lysed with 8 M urea, 75 mM NaCl, and 50 mM HEPES in the presence of protease inhibitor cocktail (Roche) and phosphatase inhibitor PhosSTOP (Roche). Protein extracts (20–50 μg) were boiled, subjected to 10% SDS-PAGE, and transferred to nitrocellulose membranes (Merck KGaA). The membranes were blocked with 5% nonfat dry milk (Bio-Rad) and incubated with specific primary antibodies at 4°C overnight. The information for the primary antibodies is listed in Supplemental Table 2. The HRP-conjugated secondary antibodies were used at 1:5,000 dilutions for 1.5 hours at room temperature. Signals were detected using ECL HRP substrate (Thermo Fisher Scientific).

### MPN patient studies.

For the peripheral blood samples from patients with MPN, mononuclear cells were purified from the peripheral blood of patients with MPN following informed consent under IRB-approved protocols at Northwestern University. Peripheral blood from the patients was red-cell lysed, followed by the collection of mononuclear cells. These cells were lysed to extract total protein for Western blotting and qPCR assays.

### IP and GST pulldown assay.

For IP assays, HEK293T cells transfected with Flag-PPIL2 or V5-p53, HUDEP-2 cells infected with V5-p53 lentivirus, or mouse erythroid cells (Ter119^+^ cells) were washed with ice-cold PBS and lysed in IP buffer (50 mM Tris-HCl [pH 8.0], 150 mM NaCl, 1% Triton X-100, 1 mM PMSF, protease inhibitor cocktail [Roche], and PhosSTOP). For denaturing IP assays, HEK293T cells transfected with V5-p53, HA-Ub, with or without Flag-PPIL2 were washed with ice-cold PBS and lysed with 8 M urea, 75 mM NaCl, and 50 mM HEPES in the presence of protease inhibitor cocktail and phosphatase inhibitor PhosSTOP. A total of 1 mg of HEK293T or HUDEP-2 cell lysates were incubated with 10 μL anti-Flag or anti-V5 magnet beads at 4°C overnight with rotation. Then, 1 mg of mouse erythroid cell lysates were incubated with 10 μL PPIL2 antibody or IgG antibody at 4°C overnight with rotation, followed by incubation with 20 μL protein A/G beads (Bimake) and incubation at room temperature for 30 minutes. Precipitated complexes were washed 3 times with IP buffer (50 mM Tris-HCl [pH 8.0], 150 mM NaCl, 1% Triton X-100, 1 mM PMSF, protease inhibitor cocktail [Roche], and PhosSTOP). Next, 5% of the sample was used as inputs and then eluted from complexes by 50 μL of 1× Laemmli sample buffer (Bio-Rad). Last, 5% of the sample inputs, 5% of the unbound fractions, and the entire eluates from the IPs were subjected to SDS-PAGE and analyzed by Western blotting analysis.

For the GST pulldown assay, 100 μg recombinant GST-PPIL2-U-box or GST-PPIL2-PPI domain were incubated with 100 μg recombinant His-p53 and 25 μL MagneGST glutathione beads (Promega) overnight at 4°C. In a separate experiment, 100 μg His-p53 domain proteins (His-p53-[1-97], [98-292], [293-393]) were incubated with 100 μg GST-PPIL2 and 25 μL MagneHis beads (Promega) overnight at 4°C. After extensive washes with TENT buffer (1% Triton X-100, 140 mM NaCl, 2 mM EDTA, and 20 mM Tris pH 8.0), the bound proteins were eluted from beads by 50 μL of 1× Laemmli sample buffer and subjected to SDS-PAGE and Western blotting.

### Luciferase reporter assay.

An 815 bp fragment (bases 79−893) of human *PPIL2* promoter was amplified by PCR and ligated into a pNL1.1 luciferase reporter vector (Promega). Next, 1 × 10^6^ HEK293T cells were cotransfected with 0.5 μg reporter vector (pNL1.1-*PPIL2* promoter), 0.02 μg pGL-4.53 vector, and 0.5 μg MAC-STA5A or empty vector control per well using TransIT-LT1 transfection reagent (Mirus) according to the manufacturer’s protocols. After 24 hours of transfection, cells were lysed with passive lysis buffer (Promega), and the expression of the reporter gene was assessed using the dual-luciferase reporter assay system (Promega).

### DNA pulldown.

pNL1.1-*PPIL2* or mutant promoter DNA labeled with biotin on the 5′-flanking region was cloned from recombinant promoter plasmids by PCR amplification. After DNA purification (QIAGEN), biotin-tagged promoter DNA (5 μg) was mixed with extracts isolated from cultured human erythroid cells (500 μg) on day 9 and 100 μL of streptavidin-coupled Dynabeads (Thermo Fisher Scientific) in binding buffer (50 mM Tris-HCl, pH 8.0, and 150 mM NaCl, 1% Triton X-100, and 1 mM phenylmethylsulfonyl fluoride) and incubated at 4°C overnight. Following incubation, the precipitated complexes were washed 3 times with washing buffer (0.5 M NaCl, 20 mM Tris pH 7.5, 1 mM EDTA). Last, 20 μL binding proteins were denatured and subjected to Western blotting assays.

### ChIP-qPCR assay.

ChIP was performed using a SimpleChIP Enzymatic Chromatin IP Kit (Cell Signaling Technology, 9002) according to the manufacturer’s protocol. Crossed chromatin (10–15 μg) was immunoprecipitated with 3 μg anti-Stat5 (Cell Signaling Technology, 25656) or anti-histone H3 antibody (Cell Signaling Technology, 4620). An equal amount of normal rabbit IgG (Cell Signaling Technology, 2729) was used as the control. The recovered DNA was analyzed by real-time PCR using the primers listed in Supplemental Table 2. Signals obtained from each IP are presented as the percentage of the total input chromatin.

### Bone marrow transplantation.

The bone marrow transplantation experiments were performed as described (15, 16, 46, 48, 49, 51). Briefly, bone marrow cells from Jak2^V617F^-knockin mice (CD45.2^+^, 8–10 weeks old) were extracted, and 2 × 10^6^ total bone marrow cells were transplanted into lethally irradiated (10 Gy) recipient mice (CD45.1^+^). For Ppil2 knockout bone marrow transplantation, HSPCs were purified from the bone marrow of WT, Jak2^V617F^-knockin mice, or p53 knockout mice (6–8 weeks old) using a c-Kit^+^ selection kit (StemCell Technologies) and cultured in StemSpan (StemCell Technologies) supplemented with 10 ng/mL murine IL-3 (StemCell Technologies), 10 ng/mL mouse IL-6 (StemCell Technologies), 50 ng/mL murine SCF, and 10 μg/mL human low-density lipoprotein (StemCell Technologies). On the second day, 80 million CRISPR-Ppil2 sgRNA or control sgRNA lentivirus were used to infect 0.5 million HSPCs with 8 μg/mL polybrene and centrifuged at 500 *g* for 1 hour at 37°C. After 12 hours of infection, cells were washed with PBS and cultured in a new complete medium for 6 hours. The cells (1 × 10^6^) were then transplanted into lethally irradiated (9.5 Gy) recipient mice (CD45.1-positive). Recipient mice were kept on antibiotic water (1.1 mg/mL neomycin and 2,000 U/mL polymyxin B, MilliporeSigma) for 3 weeks, followed by regular water.

### Bone marrow organoids derived from iPSCs.

The human bone marrow organoid differentiation was previously described (32, 33). In brief, iPSCs were purchased from StemCell Technologies (SCTi003-A). Cells were passaged as clumps using EDTA at 0.02% in PBS. The cells were then thawed and passaged for differentiation or maintenance in StemFlex (Thermo Fisher Scientific) supplemented with RevitaCell (Thermo Fisher Scientific). For differentiation, approximately 5–30 passages of iPSCs were dissociated using EDTA when colonies were approximately 100 μm in diameter. In phase I (days 0–3), cells were plated in a 6-well ULA plate and cultured in APEL2 (StemCell Technologies) supplemented with BMP4, FGF2, and VEGF-165 (50 ng/mL). In phase II (days 3–5), the cells were supplemented with BMP-4, FGF2, and VEGFA (50 ng/mL); human SCF; and Fms-like tyrosine kinase-3 ligand (Flt3) (25 ng/mL). In phases III (days 5–12) and IV (>12 days), day 5 cells were collected by gravitation for hydrogel embedding for 2 hours according to the manufacturer’s instructions. Fully polymerized gels with cell aggregates were then supplemented with VEGFA at either 50 or 25 ng/mL, VEGFC at 50 or 25 ng/mL, FGF2, BMP4, hSCF, Flt3, EPO, thrombopoietin, GM-CSF (25 ng/mL), IL-3, and IL-6 (10 ng/mL). Cells were plated in a 12-well plate until mature (~18 days). The medium was replenished every 72 hours.

For the engraftment of donor HSPCs in the iPSC-derived bone marrow organoids, CD34^+^ HSPCs purified from the bone marrow of patients with MPN were stained with CellTrace Far Red (Sigma-Aldrich) as indicated by the manufacturer. Briefly, cells were washed with PBS and resuspended at 1 × 10^6^ cells/mL in a staining solution (CellTrace Far Red 2 μM in PBS). Cells were incubated in staining solution for 30 minutes at 37°C. After incubation, CellTrace was quenched with 5 volumes of PBS with FBS (10%), spun down, and resuspended in the appropriate media. Stained cells were then treated with DMSO or different concentrations of CsA, seeded in organoids, and cultured for 3 days. The organoids were then collected for immunofluorescence staining and flow cytometry.

For immunofluorescence assays, the organoids were fixed with a 4% formaldehyde solution (Thermo Fisher Scientific) and blocked using a solution containing 2% goat serum and 1% BSA (Sigma-Aldrich), followed by incubation with primary antibodies diluted in 1% BSA at 4°C overnight. After washing with 1× PBS, the organoids were labeled with AlexaFluor conjugated secondary antibodies at 4°C overnight. The blocking solution for the entire organoid was supplemented with Triton X-100, Tween 20, and sodium deoxycholate. Sample preparation for imaging began with clearing the organoids using a fructose-glycerol (Sigma-Aldrich) clearing solution and incubating at room temperature for 20 minutes. Organoids were placed between slides prepared with approximately 3 layers of 1 cm-long pieces of sticky tape on both sides, followed by the careful placement of a coverslip on top. Confocal microscopy was performed using a Nikon AXR confocal microscope. Confocal images were acquired as representative *Z*-stacks and as maximum intensity projections (Fiji).

For flow cytometry assays, organoids were collected by sedimentation in a 15 mL Falcon tube, washed twice with 1× PBS, and then dissociated in 0.25% collagenase type I buffer (Sigma-Aldrich) at 37°C for 1 hour, followed by trituration and a further 5-minute incubation. The dissociation reaction was halted by the addition of PBS supplemented with 10% FBS. After another wash with 1× PBS, the cells were resuspended in PBS containing 0.5% BSA and 2 mM EDTA and immunostained with the following antibodies: CD11b-PE-Cy7 (BioLegend, 101216), CD3-FITC (BioLegend, 344803), CD34-BV421 (BioLegend, 343609), and CD235-PE (BioLegend, 306603).

### Statistics.

Results are expressed as the mean ± SEM unless otherwise indicated. Statistical comparisons between 2 groups were performed with a 2-tailed, unpaired Student’s *t* test, and the comparison among multiple groups was evaluated with 1-way ANOVA tests using GraphPad Prism, version 9.0 (GraphPad Software). A *P* value *of* less than 0.05 was considered statistically significant.

### Data availability.

Values for all data points in graphs are reported in the Supporting Data Values file. Data are available upon request.

## Author contributions

PW, XH, KR, EL, HB, IA, YL, MS, and JY performed the experiments and interpreted data. PW, XH, and PJ designed the experiments, interpreted data, and wrote the manuscript.

## Supplementary Material

Supplemental data

Unedited blot and gel images

Supplemental table 1

Supplemental table 2

Supporting data values

## Version 1 05/08/2025

In-Press Preview

## Version 2 07/01/2025

Electronic publication
