# Supplementary material for: PPIL2 is a target of the JAK2/STAT5 pathway and promotes myeloproliferation via degradation of p53
Source: J Clin Invest. 2025 May 8;135(13):e181394. doi: 10.1172/JCI181394 (PMC12208539; doi:10.1172/JCI181394)
Supplement: Supplemental data [file jci-135-181394-s071.pdf]

Supplemental Figures and Legend

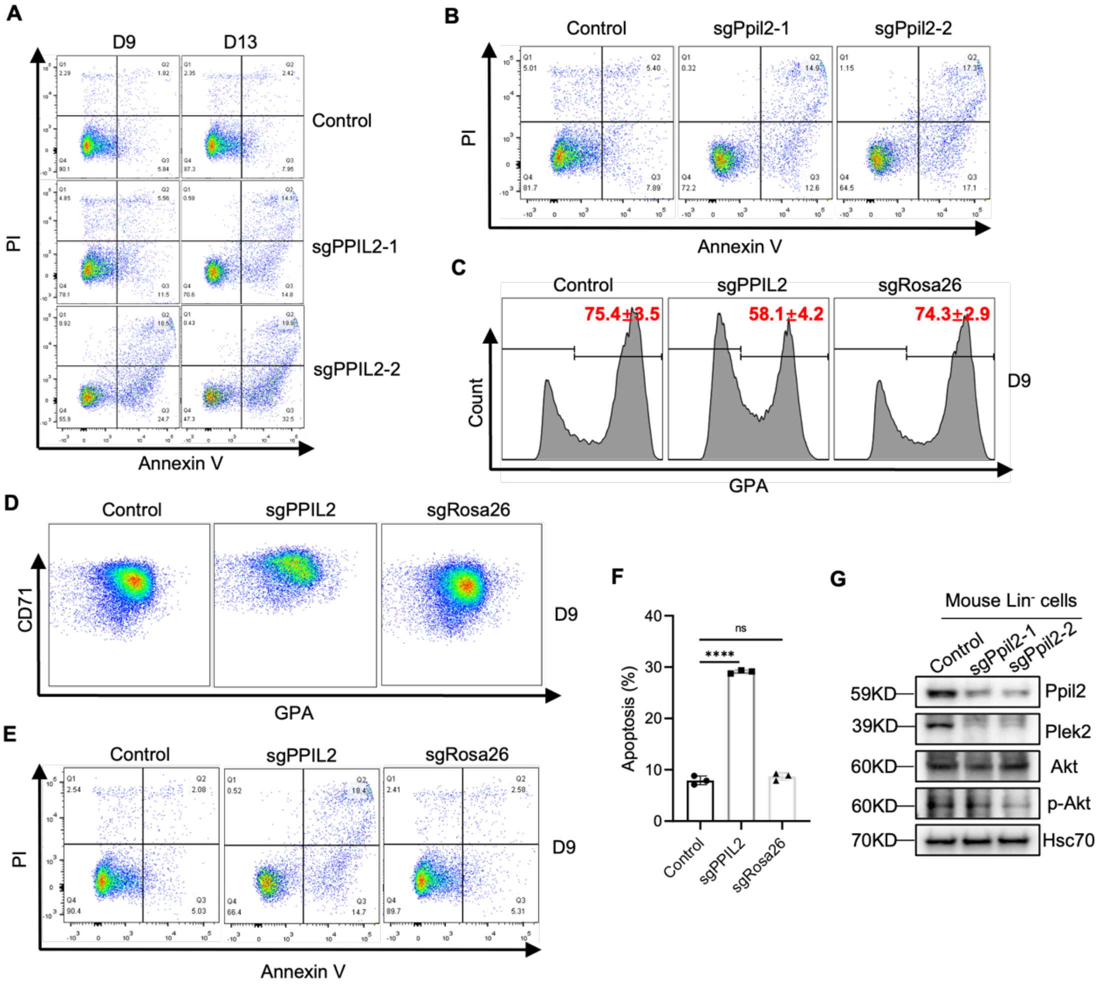

Supplemental Figure 1. PPIL2 is important for terminal erythropoiesis in vitro. A, Representative images of the flow cytometry analyses of apoptosis on day 9 and day 13 of EPO medium-cultured human CD34+ cells transduced with control or CRISPR-PPIL2 sgRNAs. **B,** Representative images of the flow cytometry analyses of apoptosis on EPO medium-cultured mouse lineage negative cells transduced with control or CRISPR-Ppil2 sgRNAs. **C,** Flow cytometric analysis of GPA (CD235a, glycophorin A) on day 9 of EPO medium-cultured CD34+ cells transduced with control, CRISPR-PPIL2 sgRNAs, or CRISPR-Rosa26 sgRNAs. **D,** Flow cytometric analyses of GPA and CD71 on cells on day 9 cultured cells as in C. **E,** Representative images of the flow cytometry analyses of cell apoptosis on day 9 cultured cells as in C. **F**, Quantification of E. **G**, Western blotting analyses of indicated proteins in EPO medium-cultured mouse lineage negative cells transduced with control or CRISPR-Ppil2 sgRNAs. Hsc70 was used as a loading control. The comparison among multiple groups was evaluated with 1-way ANOVA tests (F). ns: non-significant, **** p<0.0001.

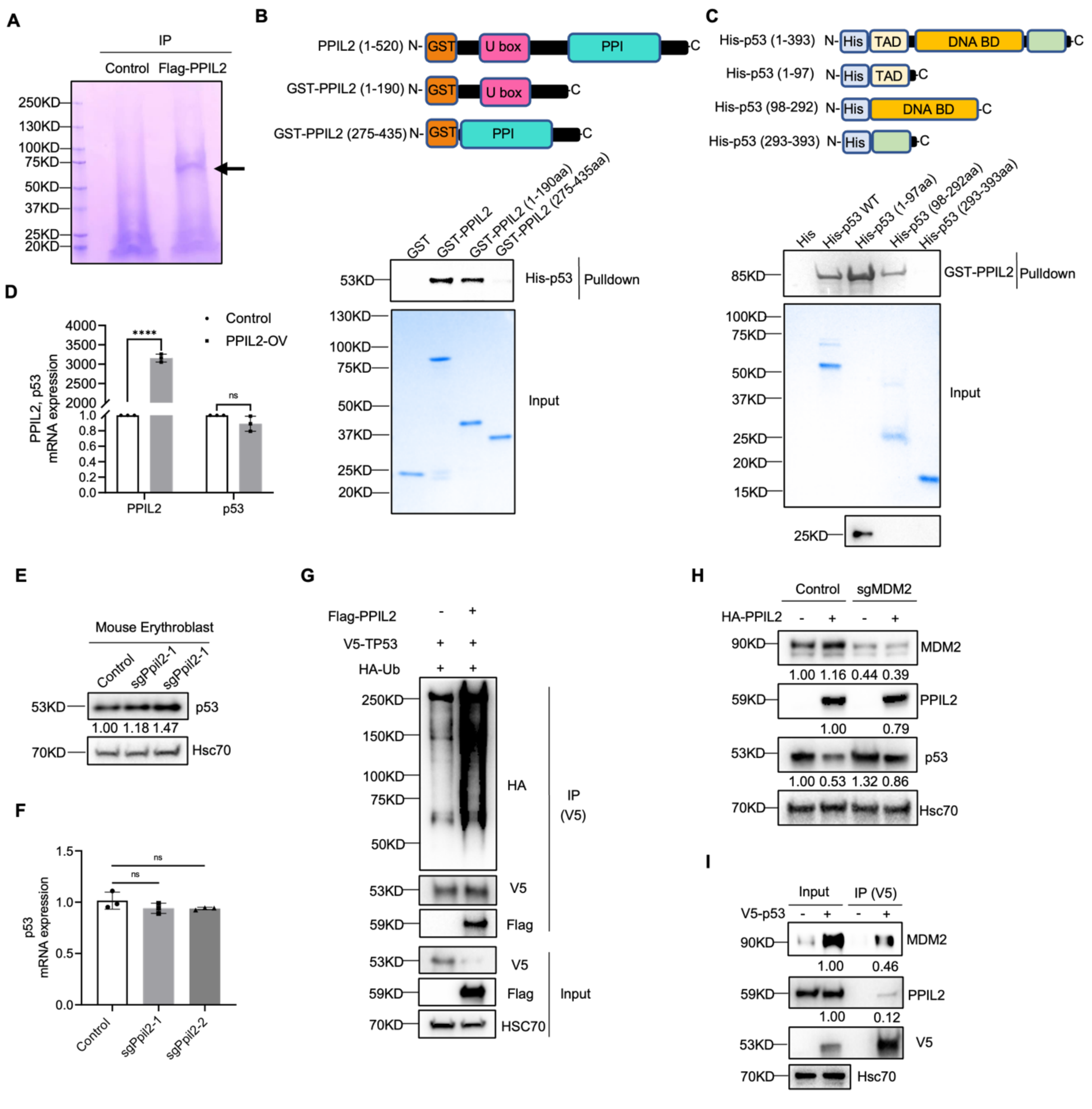

Supplemental Figure 2. PPIL2 binds to p53 and upregulates its protein level. A, Coomassie stain of 293T cell lysate following Flag-PPIL2 immunoprecipitation. The arrow indicates a prominent band in the PPIL2 precipitate. **B**, Schematic diagram of the functional domains of PPIL2 (top). GST pulldown assay using indicated recombinant GST-PPIL2 mutants with His-p53 (bottom). **C**, Schematic diagram of the functional domains of p53 (top). GST pulldown assay using recombinant GST-PPIL2 with indicated His-p53 mutants (bottom). **D**, Quantitative PCR of *PPIL2* and *TP53* expression in 293T cells transfected with Flag-PPIL2 (PPIL2-OV). **E-F**, Western blotting (E) and quantitative PCR (F) analyses of p53 expression in EPO medium-cultured mouse lineage negative cells transduced with control or CRISPR-Ppil2 sgRNA. Hsc70 was used as a loading control. **G**, Western blotting of indicated proteins after incubating anti-V5-coated beads with denatured lysates from 293T cells transfected with indicated constructs. **H**, Western blotting

analysis showing expression levels of MDM2, PPIL2, and p53 in control or MDM2 knockout 293T cells that were transfected with control or HA-PPIL2 overexpressed plasmids. HSC70 was used as a loading control. **I**, Immunoprecipitation of anti-V5 with cell lysate from 293T cells transduced with control or V5-p53 followed by Western blotting of indicated proteins. The comparison among multiple groups was evaluated with 1-way ANOVA tests (F). ns: non-significant, **** p<0.0001.

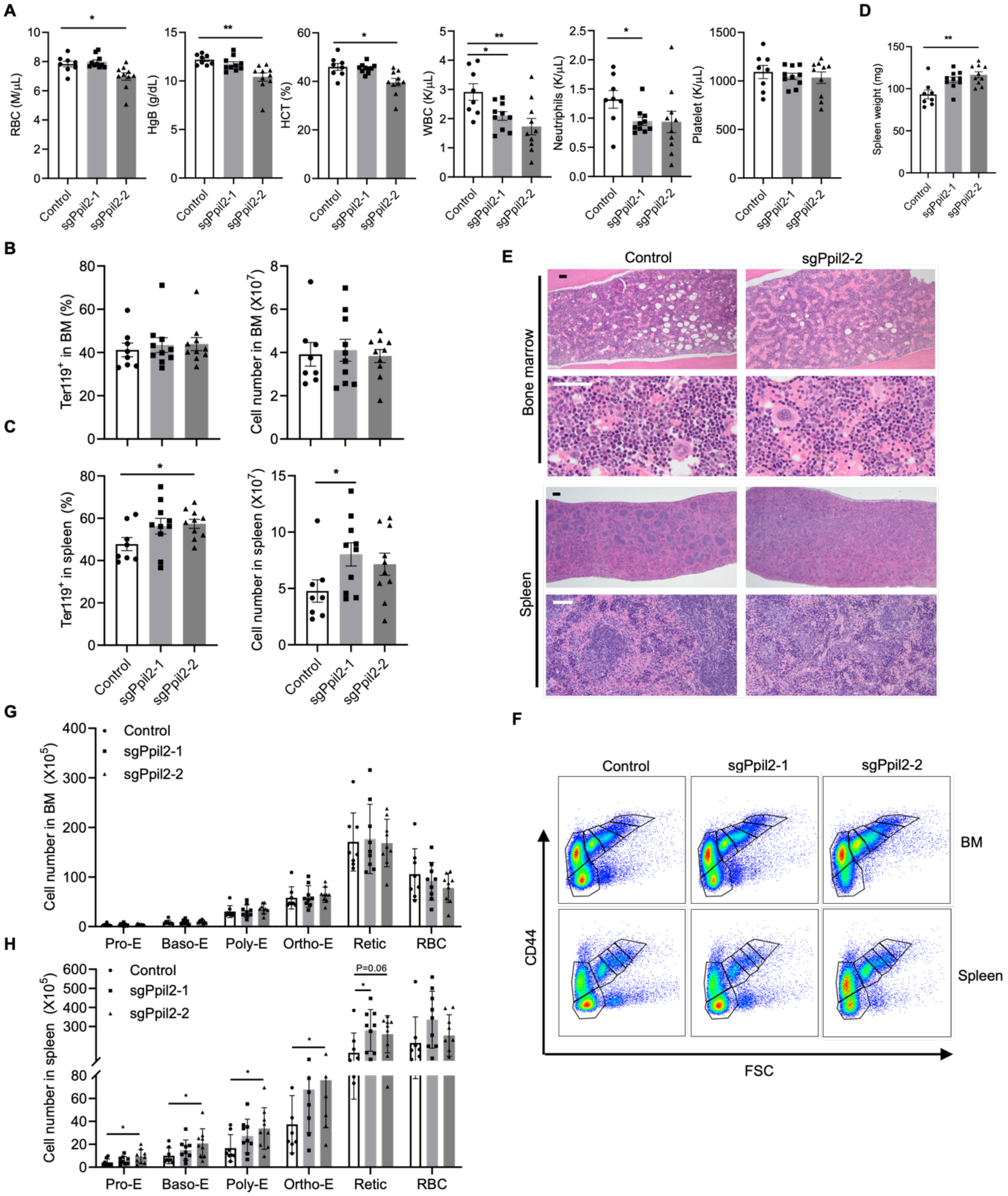

Supplemental Figure 3. Loss of Ppil2 in mouse hematopoietic cells in vivo leads to mild anemia. A, Complete blood count of mice 3 weeks after transplantation with HSPCs transduced with Cas9 and indicated sgRNAs. Control: n=8, sgPpil2-1: n=10, sgPpil2-2: n=10. **B-C**, Quantification of Ter119-positive cells by flow cytometric analyses from bone marrow (B) and spleen (C) in mice from A. **D**, Quantification of spleen weight of recipient mice from A. **E**, H&E staining of bone marrow and spleens of the indicated mice from A. Scale bars: 100 μm. **F**, Representative flow cytometric analyses of terminal erythropoiesis using CD44 and forward scatter as markers. Populations I-VI represent proerythroblasts, basophilic erythroblasts, polychromatic erythroblasts, orthochromatic erythroblasts, late orthochromatic and reticulocytes, and mature red blood cells, respectively. **G-H**, Quantification of different stages of erythroblasts from bone marrow (top) and spleen (bottom) in mice from A. Pro-E, proerythroblasts; Baso-E, basophilic erythroblasts; Poly-E, polychromatic erythroblasts; Ortho-E, orthochromatic erythroblasts; Retic, reticulocytes; RBC, red blood cells. The comparison among multiple groups was evaluated with 1-way ANOVA tests (A-D, G, F). *p<0.05, **p<0.01.

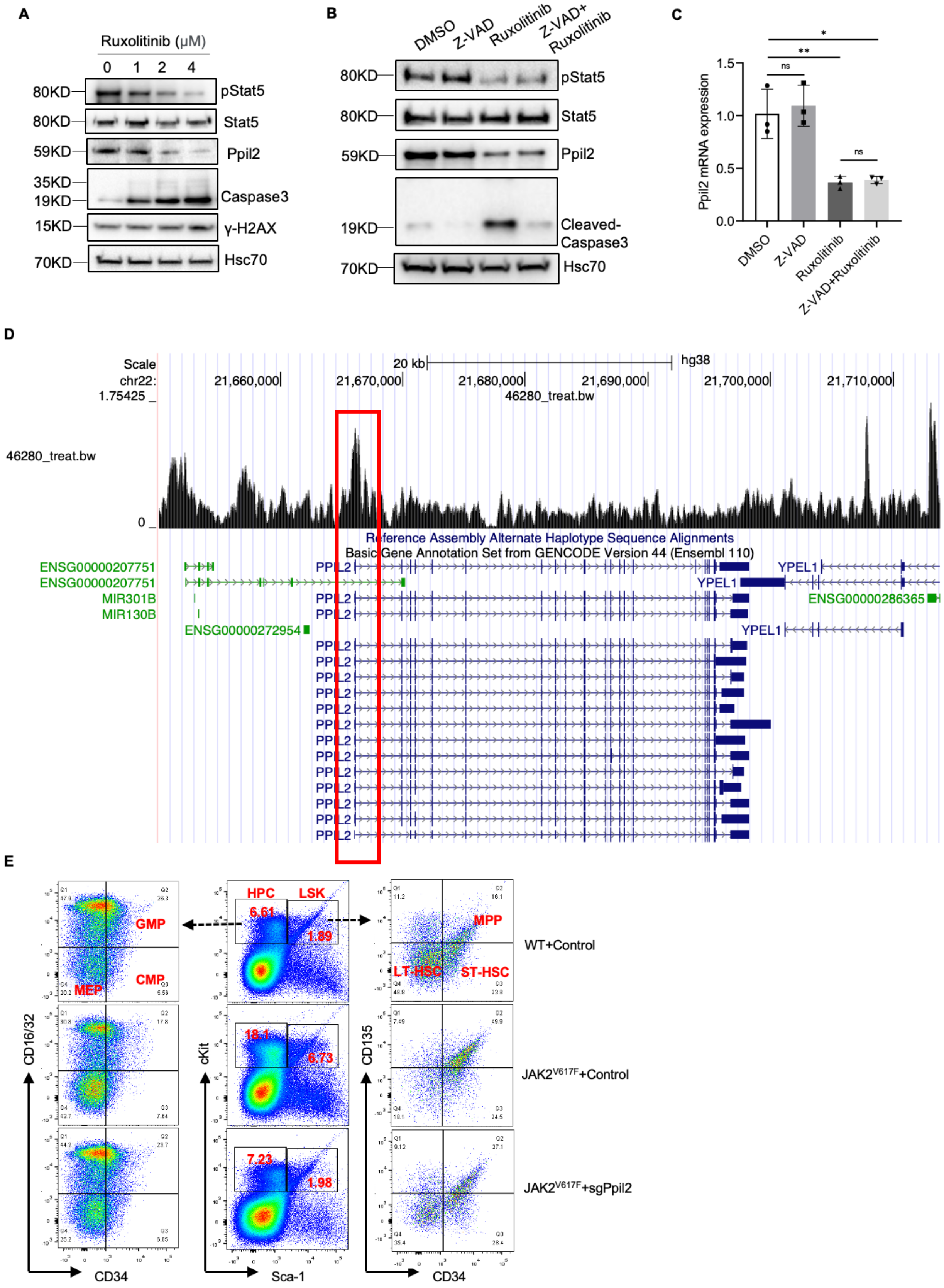
A
Ruxolitinib (μM)
0 1 2 4
80KD pStat5
80KD Stat5
59KD Ppil2
35KD 19KD Caspase3
15KD γ-H2AX
70KD Hsc70
B
DMSO Z-VAD Ruxolitinib Z-VAD+ Ruxolitinib
80KD pStat5
80KD Stat5
59KD Ppil2
19KD Cleaved-Caspase3
70KD Hsc70
C
Ppil2 mRNA expression
1.5 1.0 0.5 0.0
ns ** * ns
DMSO Z-VAD Ruxolitinib Z-VAD+Ruxolitinib
D
Scale
chr22:
1.75425 _
20 kb
hg38
21,660,000 21,670,000 21,680,000 21,690,000 21,700,000 21,710,000
46280_treat.bw
0 _
Reference Assembly Alternate Haplotype Sequence Alignments
Basic Gene Annotation Set from GENCODE Version 44 (Ensembl 110)
ENSG00000207751
ENSG00000207751
MIR301B
MIR130B
ENSG00000272954
ENSG00000286365
PPIL2
YPEL1
E
HPC LSK
GMP MEP CMP
MPP LT-HSC ST-HSC
6.61 1.89
18.1 6.73
7.23 1.98
WT+Control
JAK2V617F+Control
JAK2V617F+sgPpil2
CD16/32 CD34
cKit Sca-1
CD135 CD34

Supplemental Figure 4. A, Western blotting analyses of indicated proteins in the cultured mouse lineage negative cells in EPO medium and treated with indicated concentrations of ruxolitinib for 20 hours. Hsc70 was used as a loading control. **B-C**, Western blotting (B) and quantitative PCR (C) analyses of indicated proteins in EPO medium-cultured mouse lineage negative cells treated with DMSO, 10 μM Z-VAD-FKM, 1 μM ruxolitinib, or 10 μM Z-VAD-FKM and 1 μM ruxolitinib for 20 hours. Hsc70 was used as a loading control. **D,** STAT5A ChIP-Sequencing data showing the STAT5A binding peak on the *PPIL2* promoter region**.** Data is from Gertz J, et al. Mol Cell. 2013 Oct 10;52(1):25-36. **E,** Representative flow cytometric analyses of HSPCs from the bone marrow of mice transplanted with wild type (WT) or Jak2^{V617F} mice transduced with control or CRISPR-Ppil2 sgRNA. HPC: Lineage negative, c-Kit+ hematopoietic progenitor cells. GMP: granulocyte-macrophage progenitor. CMP: common myeloid progenitor. MEP: megakaryocyte-erythrocyte progenitor. LSK: Lin-Sca1+cKit+ cells. LT-HSC: long-term HSC. ST-HSC: short-term HSC. MPP: multipotential progenitor. The comparison among multiple groups was evaluated with 1-way ANOVA tests (C). *p<0.05, **p<0.01, ns: non-significant.

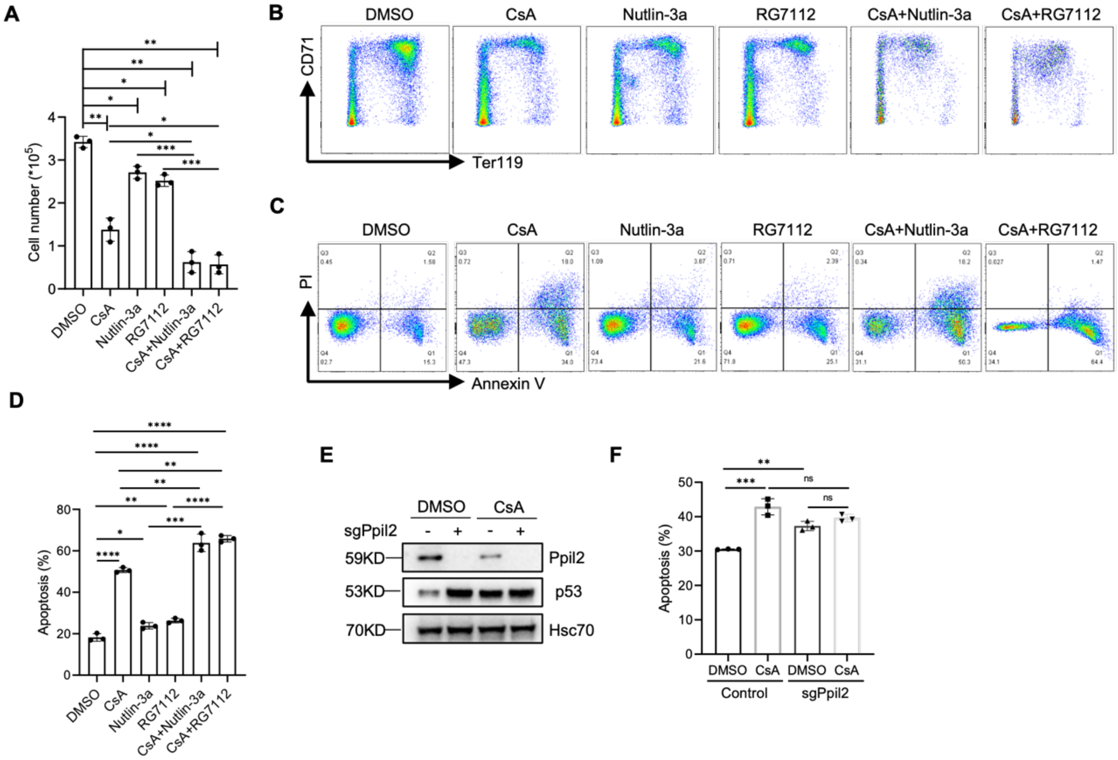

Supplemental Figure 5. Cyclosporin A inhibits erythropoiesis in vitro. A, 5 μM Nutlin-3a and RG7112 alone or in combination with 7.5 μM CsA were added to the cultured day 1 mouse erythroblasts in the presence of EPO (2 U/mL). Cells were counted on day 2. **B-C**, Representative images of the flow cytometry analyses of cell differentiation (B) and apoptosis (C) of day 2 erythroblasts. **D**, Quantification of C. **E,** Lineage-negative cells from Jak2^{V617F} knockin mice were transduced with control or CRISPR-Ppil2 sgRNA, followed by treating with DMSO or 20 μM CsA for 24 hours. The levels of Ppil2 and p53 were detected by Western blotting. Hsc70 was used as a loading control. **F,** Quantification of cell apoptosis by flow cytometry from E. The comparison among multiple groups was evaluated with 1-way ANOVA tests (A, D, F). ns: non-significant, * p<0.05, **p<0.01, ***p<0.001, and **** p<0.0001.
