# Supplementary figures and images for: PPIL2 is a target of the JAK2/STAT5 pathway and promotes myeloproliferation via degradation of p53
Source: J Clin Invest. 2025 May 8;135(13):e181394. doi: 10.1172/JCI181394 (PMC12208539; doi:10.1172/JCI181394)

Full unedited membranes:

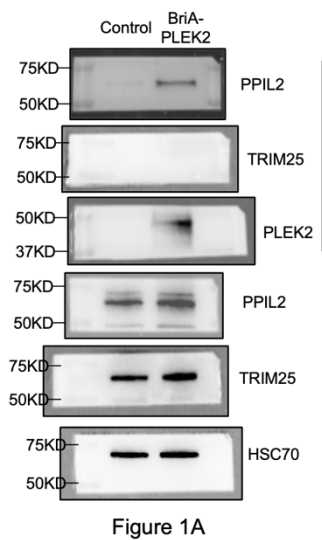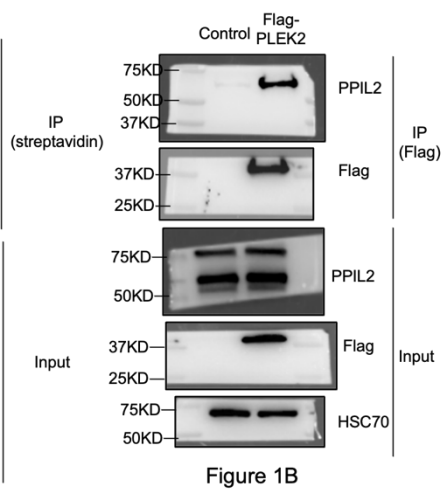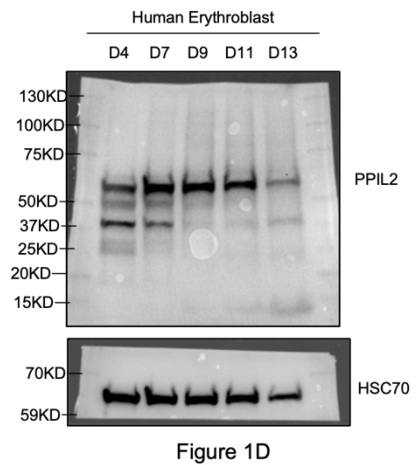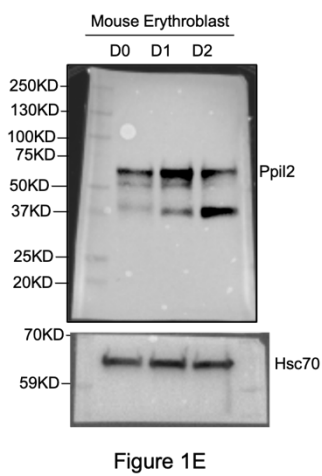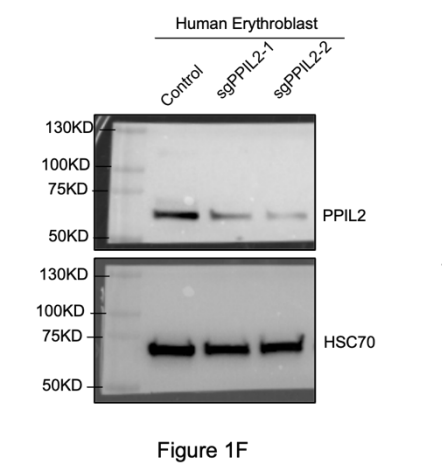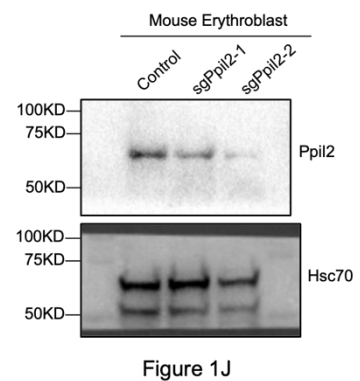

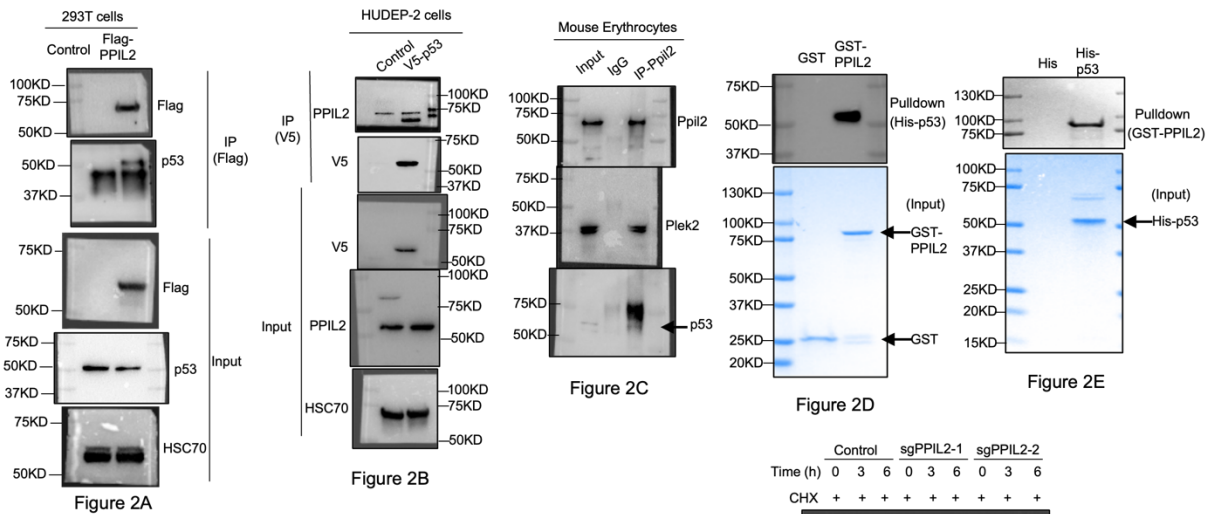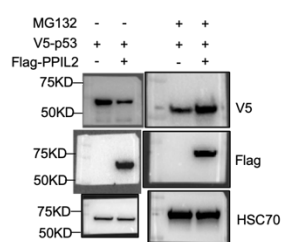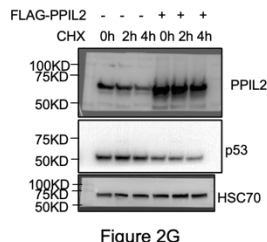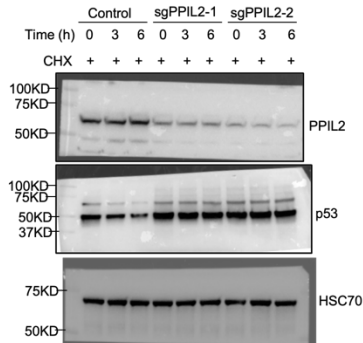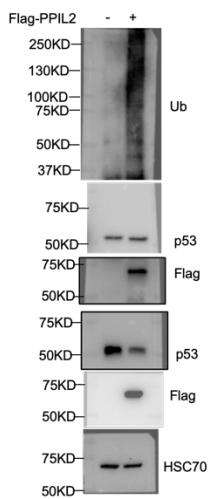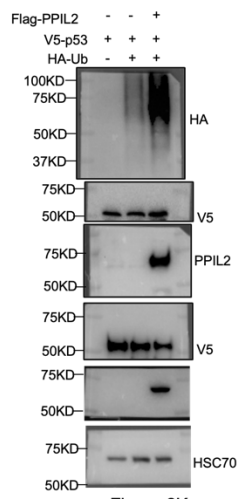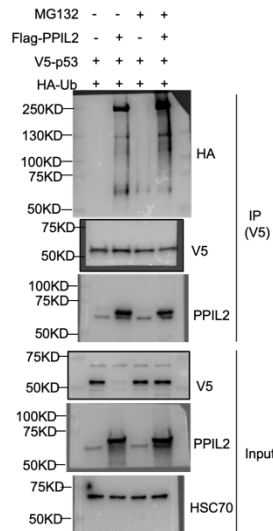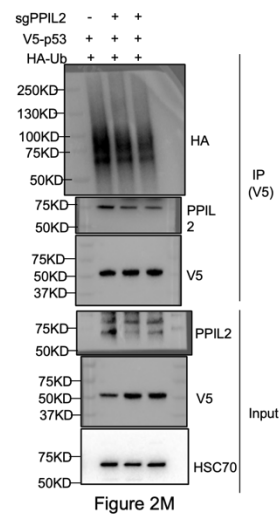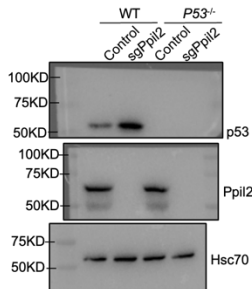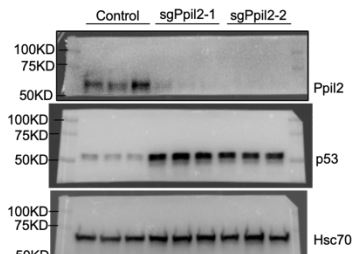

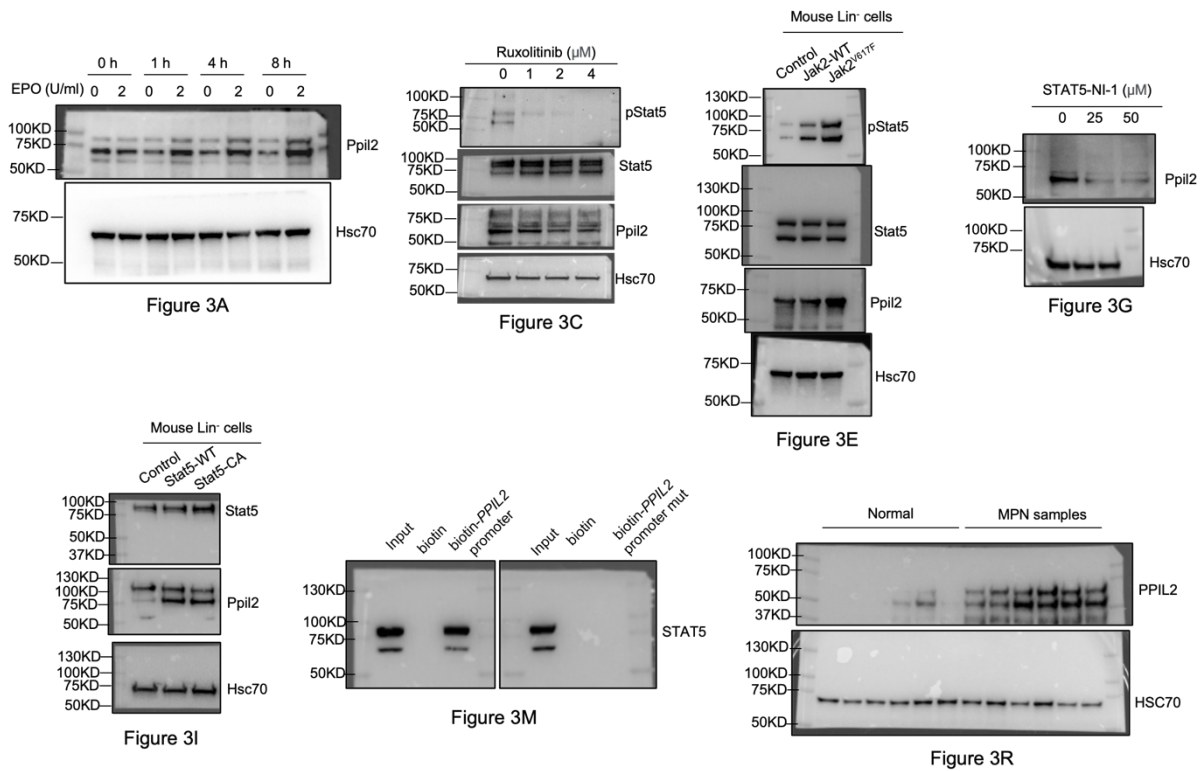

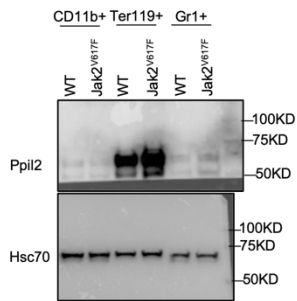

Figure 4A

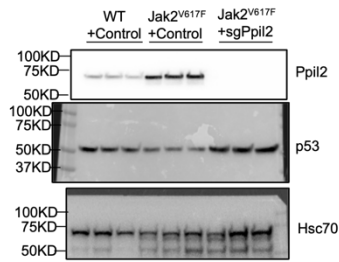

Figure 4E

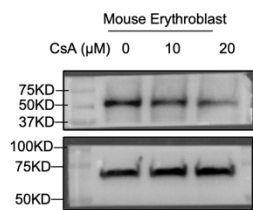

Figure 5C

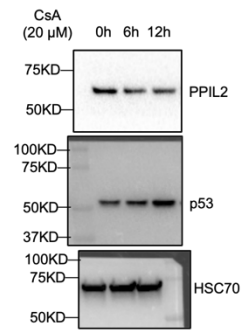

Figure 5D

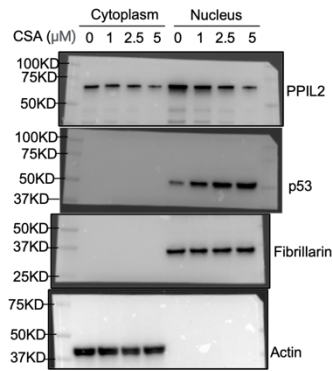

Figure 5E

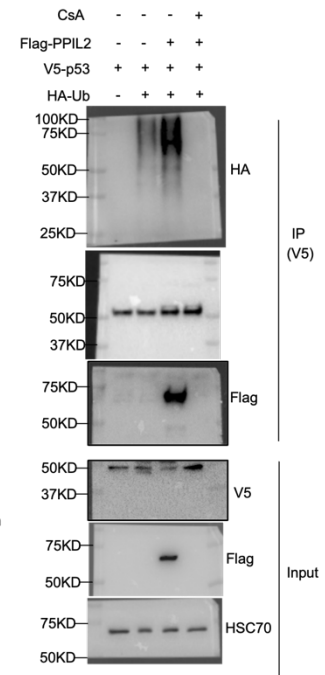

Figure 5F

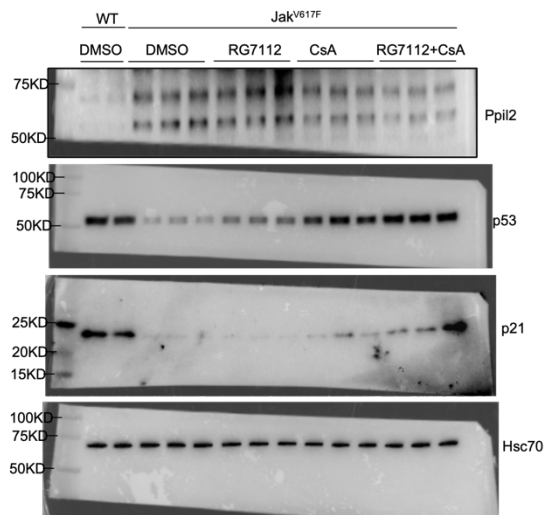

Figure 6B

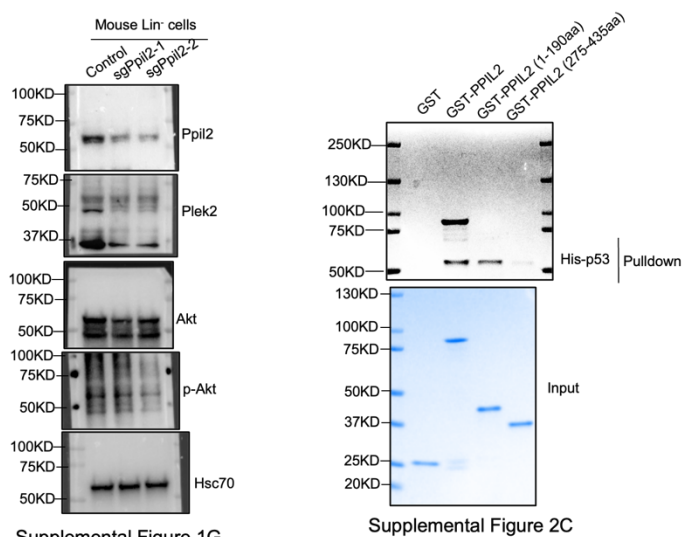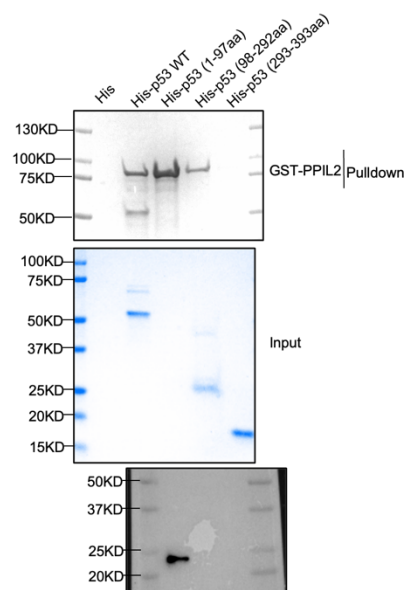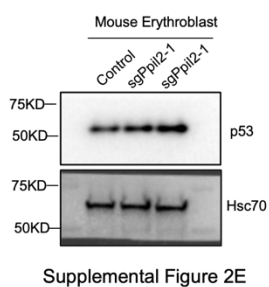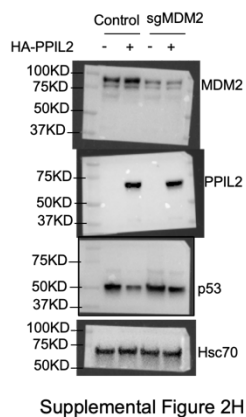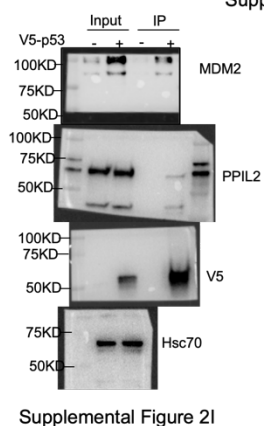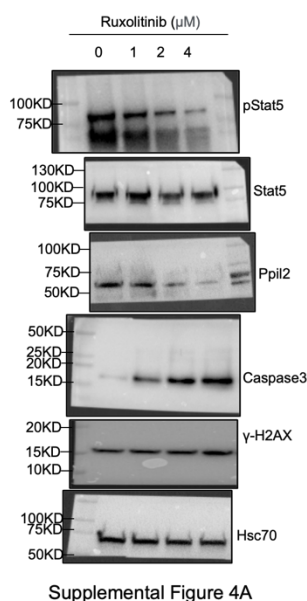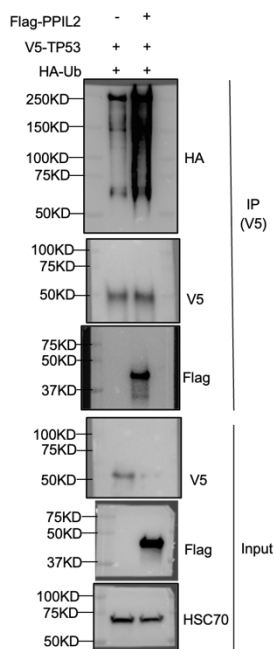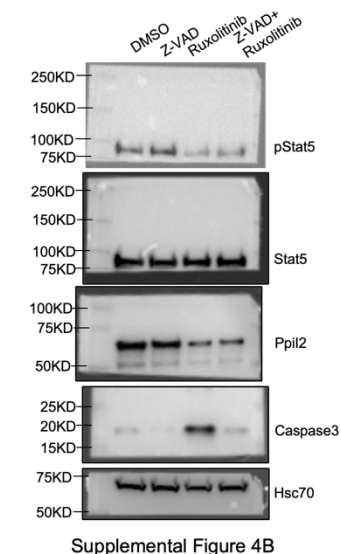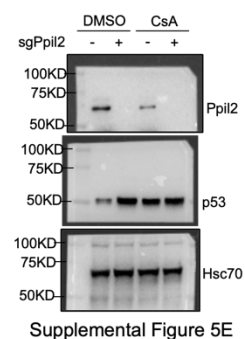

Supplement: Unedited blot and gel images [file jci-135-181394-s072.pdf]
